# Development of Chloroplast and Nuclear DNA Markers for Chinese Oaks (*Quercus* Subgenus *Quercus*) and Assessment of Their Utility as DNA Barcodes

**DOI:** 10.3389/fpls.2017.00816

**Published:** 2017-05-19

**Authors:** Jia Yang, Lucía Vázquez, Xiaodan Chen, Huimin Li, Hao Zhang, Zhanlin Liu, Guifang Zhao

**Affiliations:** ^1^Key Laboratory of Resource Biology and Biotechnology in Western China, Ministry of Education, College of Life Sciences, Northwest UniversityXi'an, China; ^2^Biology Department, University of Illinois at SpringfieldSpringfield, IL, United States

**Keywords:** plastid marker, nuclear gene, species discrimination, hybridization, phylogeny, *Quercus*

## Abstract

Chloroplast DNA (cpDNA) is frequently used for species demography, evolution, and species discrimination of plants. However, the lack of efficient and universal markers often brings particular challenges for genetic studies across different plant groups. In this study, chloroplast genomes from two closely related species (*Quercus rubra* and *Castanea mollissima*) in Fagaceae were compared to explore universal cpDNA markers for the Chinese oak species in *Quercus* subgenus *Quercus*, a diverse species group without sufficient molecular differentiation. With the comparison, nine and 14 plastid markers were selected as barcoding and phylogeographic candidates for the Chinese oaks. Five (*psb*A-*trn*H, *mat*K-*trn*K, *ycf*3-*trn*S, *mat*K, and *ycf*1) of the nine plastid candidate barcodes, with the addition of newly designed ITS and a single-copy nuclear gene (SAP), were then tested on 35 Chinese oak species employing four different barcoding approaches (genetic distance-, BLAST-, character-, and tree-based methods). The four methods showed different species identification powers with character-based method performing the best. Of the seven barcodes tested, a barcoding gap was absent in all of them across the Chinese oaks, while ITS and *psb*A-*trn*H provided the highest species resolution (30.30%) with the character- and BLAST-based methods, respectively. The six-marker combination (*psb*A-*trn*H + *mat*K-*trn*K + *mat*K + *ycf*1 + ITS + SAP) showed the best species resolution (84.85%) using the character-based method for barcoding the Chinese oaks. The barcoding results provided additional implications for taxonomy of the Chinese oaks in subg. *Quercus*, basically identifying three major infrageneric clades of the Chinese oaks (corresponding to Groups *Quercus, Cerris*, and *Ilex*) referenced to previous phylogenetic classification of *Quercus*. While the morphology-based allocations proposed for the Chinese oaks in subg. *Quercus* were challenged. A low variation rate of the chloroplast genome, and complex speciation patterns involving incomplete lineage sorting, interspecific hybridization and introgression, possibly have negative impacts on the species assignment and phylogeny of oak species.

## Introduction

Chloroplast DNA (cpDNA), which is maternally inherited most angiosperm taxa, is considered a useful tool to trace demographic history, explore species divergence and identify species (Thomson et al., 2010; van Velzen et al., 2012; Greiner et al., 2014). The utility of cpDNA is highly manifested by the increasing number of phylogeographic and phylogenetic studies during recent decades (Hickerson et al., 2010; Qiu et al., 2011; Liu et al., 2012). For recently diverged plant species, uniparental inherited cpDNA with high levels of intraspecific genetic diversity and differentiation can provide data to infer population dynamics and phylogeographic patterns (Tzedakis et al., 2013). In addition, when chloroplast gene loci have diverged from a common ancestor and have evolved independently, they are expected to possess interspecific discrepancies, namely the “DNA barcode,” which are expected to be powerful tools for species discrimination, facilitating the recognition of new species as well as biodiversity assessment thus providing useful information for the development of conservation strategies (Kress et al., 2005; Hollingsworth et al., 2011; Krishnamurthy and Francis, 2012; Taylor and Harris, 2012; Li et al., 2015).

Along with traditional morphology-based taxonomy, DNA barcoding, using one or several of the proposed DNA regions to identify species, is particularly useful for taxonomists (DeSalle et al., 2005; Chase et al., 2007; CBOL Plant Working Group, 2009). In contrast to the portion of the mitochondrial Cytochrome Oxidase 1 (COI), which seems common and sufficient to fulfill almost all animal barcodes (Hebert et al., 2003), suitable barcodes to distinguish plant species include some plastid regions such as *rbc*L, *mat*K, and *trn*H-*psb*A, as well as the nuclear ribosomal internal transcribed spacer (ITS) (Kress and Erickson, 2007; CBOL Plant Working Group, 2009; China Plant BOL Group, 2011; Wang X. C. et al., 2015). Additionally, low (single)-copy nuclear loci related to strong reproductive isolation, which serve as the “speciation genes,” may also be potentially useful in species discrimination, albeit designing universal primers for the low (single)-copy nuclear genes are difficult (Wu, 2001; Chase et al., 2005; Ran et al., 2010; Wang et al., 2011). For land plants, although a number of DNA barcodes have been evaluated, special interests and challenges are still under exploration in identifying closely related or recently evolved species, especially within species-rich genera (Liu et al., 2011; Yan et al., 2015). Failures of DNA barcoding to properly discriminate recently diverged species may be due to incomplete lineage sorting or lack of a “barcode gap,” and related to large effective population size as well as low evolutionary rate of species (van Velzen et al., 2012). Additionally, insufficient sampling in a species-rich genus and a lack of consensus on suggested markers also cause inaccurate species identification among closely related species (Ran et al., 2010). Several different DNA barcoding methodologies, such as distance-based, tree-based, similarity-based, and character-based methods have been approved for the assessment of species discrimination, yet dissimilar species identification powers exist among these analytical methods based on same datasets (Zou et al., 2011; van Velzen et al., 2012; Yan et al., 2015). In general, DNA barcoding has become a well-funded, global enterprise as a rapid technique for species identification (Taylor and Harris, 2012). However, challenges still exist in the discrimination of closely related or recently diverged species groups (van Velzen et al., 2012), such as the oak lineage.

Oaks (*Quercus* L., Fagaceae) are anemophilous species with approximately 400–500 taxa widespread throughout the Northern Hemisphere, belonging to a taxonomically complex group (Dumolin-Lapegue et al., 1999; Aldrich and Cavender-Bares, 2011). The genus *Quercus* has been recognized as two major clades comprising five or six infrageneric key groups as *Lobatae* Loudon, *Protobalanus* (Trelease) Camus, *Quercus* Linneaus, *Cerris* Loudon, *Ilex* Loudon as well as the tropical to subtropical, evergreen (subgenus) *Cyclobalanopsis* Oersted group in Southeast Asia (Oh and Manos, 2008; Denk and Grimm, 2009, 2010; Denk and Tekleva, 2014; Hubert et al., 2014). However, the *Cyclobalanopsis* group is also treated as a subgenus of *Quercus* by some taxonomists due to its controversial phylogenetic position (i.e., Zhou, 1992; Nixon, 1993; Manos et al., 2001; Menitsky, 2005), and we regard it as such here with focus on the Chinese oak species in subg. *Quercus*. Using cpDNA markers, a series of phylogenetic and phylogeographical studies have mainly been concentrated on oaks distributed in Europe and North America (Petit et al., 2002; Magni et al., 2005; Grivet et al., 2006; Cavender-Bares et al., 2011; Simeone et al., 2016); but these markers are barely shared between the two major research areas. A set of 52 polymorphic cpDNA primers was proposed for the postglacial migration reconstruction of *Quercus rubra* L. (Borkowski et al., 2014), yet the universality of these markers in other oaks was unknown. To date, only two barcoding prospects have been performed on Italian and Euro-Mediterranean oaks with some of the proposed DNA barcodes (*rbc*L, *rpoC*1, *trn*H-*psb*A, *mat*K, and ITS2; Piredda et al., 2011; Simeone et al., 2013). While 12 Italian oak species studied revealed extremely low discrimination success (0%), the resolution in Euro-Mediterranean oaks investigated was up to 87.8% when a combination of plastid and nuclear (ITS2) regions was used, yet they sampled large scale ranges with limited sample size (one or two individuals) for most species. The lack of unique DNA barcodes for oaks is suggested to be the result of inconsistent and limited taxonomy, low variation rates of the barcodes, frequent interspecific hybridization, incomplete lineage sorting, shared ancestral polymorphism and reticulation (Simeone et al., 2013), which indicates challenges and the lack of efficient markers for barcoding the species-rich genus *Quercus*.

China harbors a high diversity of oaks in subg. *Quercus* with 62 species described in the *Flora of China* (the Chinese version), while the English revision retained 35 species, essentially removing the hybrids and morphologically similar species (http://www.floraofchina.org/). Based on morphological and anatomical studies of pollen and leaves (Zhou et al., 1995; Pu et al., 2002; Peng et al., 2007), the Chinese oaks (subg. *Quercus*) are divided into five morphology-based sections: Quercus, Aegilops, Heterobalanus, Engleriana, and Echinolepides. Of these five sections, the first two consist of deciduous oak species that are included in phylogenetic Groups *Quercus* and *Cerris*, while the remaining sections comprise evergreen oak species are hypothesized to be nested within Group *Ilex* (Pu et al., 2002; Denk and Grimm, 2009), although particular phylogenetic tests for the Chinese oaks are absent. Recent phylogeographic studies have been performed on some widespread deciduous species in Sections Quercus and Aegilops, suggesting that geological and climatic changes during the Neogene and/or Pleistocene have acted as potential triggers for intra- and inter-specific differentiation of these Chinese oaks (Zeng et al., 2011, 2015; Chen et al., 2012; Wang Y. H. et al., 2015; Zhang X. W. et al., 2015; Yang J. et al., 2016). Additional studies have mainly focused on ecological issues of Section Heterobalanus, which contains about nine sclerophyllous alpine species and occurs mainly in the Hengduan Mountains (Yang et al., 2009; Feng et al., 2016). Other oaks, for example the species of morphology-based Sections Engleriana and Echinolepides are mainly endemics and relics with narrow and scattered distributions, and most of them are endangered due to habitat loss. However, the species discrimination and differentiation, as well as phylogeny and evolution of the entire Chinese oak species have yet to be well uncovered due ambiguous species boundaries and lack of universal molecular markers.

In this study, over 200 individuals of 35 Chinese oak species of subg. *Quercus* (Table 1) were collected to explore species identification using DNA barcoding analyses. Particularly, to provide sufficient barcode candidates targeting the barcoding analyses, we (1) first developed universal cpDNA markers for the Chinese oak species using a comparative method between the chloroplast genomes of *Q. rubra* and *Castanea mollissima* Blume. Designed cpDNA primers were tested on a subset of 14 deciduous oaks to obtain candidate DNA barcodes. In addition, cpDNA markers generating highly intraspecific divergence were also identified for future phylogeographic studies of the Chinese oak species. We then (2) selected five of the identified cpDNA barcode candidates in step (1) with addition of the proposed ITS and a single-copy nuclear gene (SAP) to barcode the 35 Chinese oaks. Four different DNA barcoding methods were compared to evaluate the resolution rates of species identification. Finally, based on the (tree-based) barcoding results, we discussed the potential implications for phylogenetic prospects of the Chinese oaks in subg. *Quercus*.

**Table 1:** **Taxon information of 35 Chinese oaks in ***Quercus*** subgenus ***Quercus*** used in this study**.

**Group**	**Section**	**Species**	**Sample size**	**Location (Province)**
*Quercus*	Quercus	*Quercus aliena* Blume	9	Weining (Guizhou); Shaoxing (Zhejiang); Qinhuangdao (Hebei)
		*Quercus aliena* Blume var. *acutiserrata* Maximowicz ex Wenzig	9	Jiujiang (Jiangxi); Anlong (Guizhou); Emeishan (Sichuan)
		*Quercus dentata* Thunb	9	Foping (Shaanxi); Shenyang (Liaoning); Guangyuan (Sichuan)
		*Quercus fabri* Hance	9	Weining (Guizhou); Enshi (Hubei); Dongkou (Hunan)
		*Quercus serrata* Murray	8	Guangyuan (Sichuan); Dujiangyan (Sichuan); Bikou (Gansu)
		*Quercus serrata* Murray var. *brevipetiolata* (A. DC.) Nakai	6	Shangnan (Shaanxi); Jiujiang (Jiangxi)
		*Quercus liaotungensis* Koidz. (or *Quercus wutaishanica* Blume)	9	Mengda (Qinghai); Huayin (Shaanxi); Zhashui (Shaanxi)
		*Quercus mongolica* Fischer ex Ledebour	9	Xiyang (Shanxi); Changchun (Jilin); Yantai (Shandong)
		*Quercus griffithii* Hook. f. et Thoms. ex Miquel	6	Lijiang (Yunnan); Tianlin (Guangxi)
		*Quercus yunnanensis* Franchet	3	Yanyuan (Sichuan)
		*Quercus stewardii* Rehd.	3	Huoshan (Anhui)
*Cerris*	Aegilops	*Quercus acutissima* Carr.	9	Funing (Yunnan); Longnan (Gansu); Qinhuangdao (Hebei)
		*Quercus variabilis* Blume	6	Xixia (Henan); Yichang (Hubei)
		*Quercus chenii* Nakai	3	Xinning (Hunan)
*Ilex*	Heterobalanus	*Quercus spinosa* David ex Franchet	15	Hanzhong (Shaanxi); Jiulong (Sichuan); Shiyan (Hubei); Lijiang (Yunnan); Dali (Yunnan); Shangluo (Shaanxi); Xianju (Zhejiang); Hualien (Taiwan)
		*Quercus aquifolioides* Rehd. et Wils.	8	Maerkang (Sichuan); Mangkang (Tibet); Maoxian (Sichuan); Lijiang (Yunnan)
		*Quercus rehderiana* Hand. -Mazz.	3	Maoxian (Sichuan)
		*Quercus pseudosemecarpifolia* A. Camus	6	Weining (Guizhou); Luding (Sichuan); Lijiang (Yunnan)
		*Quercus pannosa* Hand. -Mazz.	4	Bomi (Tibet); Muli (Sichuan)
		*Quercus longispica* (Hand. -Mazz.) A. Camus	6	Maerkang (Sichuan); Muli (Sichuan); Lijiang (Yunnan)
		*Quercus monimotricha* Hand. -Mazz.	4	Yanyuan (Sichuan); Kangding (Sichuan)
		*Quercus senescens* Hand. -Mazz.	10	Chayu (Tibet); Lijiang (Yunnan); Muli (Sichuan); Huize (Yunnan); Weining (Guizhou)
		*Quercus guajavifolia* H. Leveille	8	Maerkang (Sichuan); Deqin (Yunnan); Lijiang (Yunnan); Muli (Sichuan)
		*Quercus semecarpifolia* Smith	3	Linzhi (Tibet)
		*Quercus gilliana* Rehd. et Wils.	3	Maoxian (Sichuan)
	Engleriana	*Quercus engleriana* Seem.	3	Tongren (Guizhou)
		*Quercus cocciferoides* Hand. -Mazz.	3	Muli (Sichuan)
		*Quercus phillyraeoides* A. Gray	6	Shaowu (Fujian); Chongqing
		*Quercus franchetii* Skan	3	Miyi (Sichuan)
		*Quercus acrodonta* Seemen	6	Xinning (Hunan); Shangnan (Shaanxi)
		*Quercus lanata* Roxb	3	Tongmai (Tibet)
		*Quercus tarokoensis* Hayata	4	Taroko (Taiwan)
	Echinolepides	*Quercus dolicholepis* A. Camus	6	Longlin (Guangxi); Enshi (Hubei)
		*Quercus oxyphylla* (E. H. Wilson) Handel-Mazzetti	6	Lueyang (Shaanxi); Shaowu (Fujian)
		*Quercus baronii* Skan	9	Linfen (Shanxi); Xian (Shaanxi); Lushi (Henan)

## Materials and methods

### Taxon information, DNA isolation and PCR amplification

A total of 35 oak species representing the five morphology-based sections (corresponding to the phylogenetic Groups *Quercus, Cerris*, and *Ilex*, see the introduction) of the Chinese oaks (Zhou et al., 1995; Pu et al., 2002; Peng et al., 2007) were collected following the morphological classification of the *Flora of China* (Table 1). The deciduous North American species *Q. rubra* was used for primer development; screening of plastid primers was conducted on a subset of 14 deciduous oaks, each with three to four individuals, including 11 species of the Section Quercus and three of Aegilops. For DNA barcoding analyses below, we used all 35 oak species with increased sample size (Table 1). Voucher specimens of the 35 Chinese oak species collected were archived in the herbarium of College of Life Sciences at Northwest University (Table S1). DNA extractions and PCR protocols were the same as in Yang J. et al. (2016).

### Development and screening of the chloroplast primers

The complete chloroplast genomes of *Q. rubra* (NC_020152.1; Alexander and Woeste, 2014) and *C. mollissima* (NC_014674.1; Jansen et al., 2011) were aligned and compared in program Mauve (Darling et al., 2010) with full alignment, iterative refinement parameters and default seed weight to search potential genetic variation. The large single copy (LSC) and small single copy (SSC) regions exhibiting gaps and variations between the two genomes were extracted and divided into suitable fragments for primer design. Locations of the extracted cpDNA fragments were identified based on the chloroplast genome of *Q. rubra*, and conserved regions flanking variable regions were used to design primers. Additionally, repetitive structures (except the mononucleotide repeats) in the chloroplast genome, which can provide additional genetic information at low taxonomic levels, were searched in program REPuter (Kurtz et al., 2001) with parameters of minimal repeat size of 8 bp and maximum repeats of 50 bp. Primers were designed using the PrimerSelect program in DNAStar software (DNASTAR Inc., Wisconsin, USA) with the following characteristics: primer length = 18–24 bp, melting temperature = 50–65°C and default parameters for the rest of the conditions. Product length was restricted to 400–1,100 bp to facilitate standard PCR amplification.

For each designed plastid marker, obtained sequences for 14 deciduous oak species (GenBank accession numbers: KX824782-KX825832) were visually checked and aligned in BioEdit 7.0.9.0 (Hall, 1999). Ambiguous sites and poly (A and T) structures were removed, while indels were retained for potential informative characters (Jaen-Molina et al., 2015). To choose potential barcoding markers for species discrimination, all alignments were visually scanned, and markers displaying species-specific characters (including gaps) in individual oak species were retained as candidate barcodes. To select universal primers that generate high intraspecific diversity for future phylogeographic studies, the mean diversity (nucleotide diversity, π) for each species was estimated using MEGA 6.06 (Tamura et al., 2013). Two criteria were used to filter the designed cpDNA markers: (1) primers could be successfully amplified among most of the 14 deciduous oaks; and (2) the estimated mean diversity of the 14 oak species (π) > 0.0005; this threshold value was chosen based on the average estimation of cpDNA nucleotide diversity documented in previous phylogeographic researches on anemophilous tree species such as *Populus balsamifera* (Breen et al., 2012), *Pteroceltis tatarinowii* (Li et al., 2012), and *Quercus variabilis* (Chen et al., 2012). Sequences having repetitive structures were evaluated with the deletion-insertion polymorphism (DIP) analysis using the Multiallelic option in DnaSP 5.0 (Rozas et al., 2003), but were only performed on two widespread species *Quercus aliena* and *Quercus acutissima*, which have sample sizes of nine individuals for each species.

### DNA barcoding analyses

Five cpDNA candidate barcodes were selected for barcoding the Chinese oak species, as a combination of them showed the maximum species identification potential among 14 Chinese deciduous oaks (see the results). Additionally, two nuclear genes with newly designed primers, the ITS region flanking ITS1-5.8S rDNA-ITS2 (Forward: GTTCGGGCGACGGGACAC; Reverse: CCTGCGGGCGGGGACCTC) and a predicted zinc finger A20 and AN1 domain-containing stress-associated protein 8-like gene (SAP) (Vij and Tyagi, 2006) (Forward: ATGGAGCATGATGAGACGGG; Reverse: GCCTTAACAACAGGGTTGGC) which was confirmed as a single-copy gene using a BLAST search based on a comparative transcriptome work (unpublished data) and against the *Quercus* EST database in the National Center for Biotechnology Information (NCBI), were joined to test their discriminatory power in subg. *Quercus* of China. All obtained DNA sequences for each candidate region (GenBank accession numbers: KX836866-KX838287) were checked visually and aligned with BioEdit 7.0.9.0. Ambiguous sites and poly (A and T) structures were removed, while informative insertion-deletion events (gaps) showing species-specific characters were coded as nucleotide substitutions following Simmons and Ochoterena (2000). Potential pseudogenes of the ITS region (Manos et al., 2001; Ma and Zhou, 2006; Denk and Grimm, 2010) were checked and filtered using multiple examinations (i.e., G+C content, secondary structure and conserved domains of ITS2, and conserved motifs in 5.8S rDNA; see Note S1); only functional ITS orthologues were retained for the barcoding analysis. Sequencing peaks of nucleotide sites having an overlap greater than 80% in the nuclear sequences were treated as heterozygous, and ambiguity was coded according to the IUPAC (International Union of Pure and Applied Chemistry). Individual barcodes and all possible combinations of the seven candidates (five plastid and two nuclear regions) were evaluated using four different barcoding methods as follows:

Method-1 Genetic distance-based: DNA barcoding gaps of the Chinese oak species were evaluated by comparing the distributions of ranked pairwise differences (p-distance) with the maximum intraspecific genetic divergence calculated in MEGA 6.06 with 1,000 bootstraps for each candidate barcode. A barcode gap would exhibit a sudden increase in the vicinity of the maximum intraspecific divergence. For each barcode candidate and all possible combinations, the obtained genetic distance matrices were then used as input to explore potential species groups using the Automatic Barcode Gap Discovery (ABGD) tool with estimated maximum intraspecific distance as threshold and 100 simulation steps (Puillandre et al., 2012). A species group was considered as successfully delimited if conspecific individuals were partitioned into the same group without sequences from other species. Differentiations between the intra- and interspecific divergences for each candidate and infrageneric partition (i.e., the five morphology-based sections of the Chinese oaks and traditional phylogenetic classification of *Quercus*) were also compared using the Student's *t*-test and Wilcoxon rank sum test in R 3.2.3 (R Development Core Team, 2011).

Method-2 BLAST-based: Sequences of the seven candidate barcodes were concatenated as a local reference database. Individual sequences from a specified barcode were then used to query the reference database with the local NCBI-blast-2.2.28+ program in C environment (Camacho et al., 2009). Query parameters were compiled as: –query BLAST_sequence –db reference_database –out BLAST_results –outfmt 7 –max_target_seqs 10. Species discrimination was considered successful when all individuals of a species had a top hit to conspecific individuals (the query sequence itself was excluded from the blast results).

Method-3 Character-based: The character-based DNA barcode method in BLOG (Barcoding with LOGic) 2.0 was used for species identification under a machine learning approach which selects a small set of suitable positions from the reference sequences to classify species (Weitschek et al., 2013). Raw sequences of each candidate barcode and combinations were used as the reference database and were tested with 100% slicing for training, 50 POSCOST and 800 NEGPOST for the logic formulas against itself to verify the identification power of each database. For each reference, the total percentage of correct species classification rates was recorded.

Method-4 Tree-based: Unrooted neighbor-joining (NJ) trees were constructed in MEGA 6.06 based on pairwise deletion and the p-distance model with 1,000 bootstrap replicates according to Yan et al. (2015) for species-level discrimination of closely related groups. Only species with multiple individuals forming a monophyletic clade in the NJ tree were considered to be successfully identified.

## Results

### Comparison of the two chloroplast genomes

The total consensus chloroplast genome length of *Q. rubra* was 161,304 and 160,799 bp for *C. mollisima* (Figure S1). Both species had similar GC contents but differed in the total number of genes with *Q. rubra* having 11 more genes than *C. mollisima*. The LSC and SSC regions of *Q. rubra* were larger (91,121 and 20,150 bp) than those of *C. mollissima* (90,432 and 18,995 bp). In contrast, the inverted repeat (IR) of *Q. rubra* was shorter than in *C. mollissima*. Searching for repetitive structures within the chloroplast genome revealed 12 repeats in *Q. rubra*, while *C. mollissima* had eight (Table 2). When the IR regions were removed, comparison of the chloroplast genome of *Q. rubra* with *C. mollissima* as reference yielded 701 plastid SNP sites and 221 gaps for primer design.

**Table 2:** **Comparison of major features of the two chloroplast genomes ***Quercus rubra*** and ***Castaena mollissima*****.

	***Quercus rubra***	***Castanea mollissima***
Total length (bp)	161,304	160,799
LSC length (bp)	91,121	90,432
SSC length (bp)	20,150	18,995
IR length (bp)	25,051	25,686
GC content (%)	36.79	36.80
Number of gene	138	127
Number of repetitive structure	12	8

### Information of designed primers for the Chinese oaks

Thirty-nine pairs of plastid primers were designed based on detected variations and gaps in the LSC and SSC regions of the chloroplast genome of *Q. rubra*. Total expected PCR product length of the 39 primers was 28,555 bp, covering 17.7% of the *Q. rubra* chloroplast genome. In addition, the repetitive structures in the chloroplast genome of *Q. rubra* yielded eight primer pairs. The average length of PCR products for these 47 primers was 710 bp. Primer screening on 14 deciduous oak species resulted in successful amplification of 30 primer pairs with an average success rate of 62% among the 47 designed markers (Table S2); most primers showed high universality across the 14 Chinese deciduous oaks.

To select suitable cpDNA markers for future phylogeographic studies, estimation of the mean genetic diversity (π) suggested that 12 primer pairs could reveal high intraspecific diversity in most of the 14 deciduous oak species based on our criteria (π > 0.0005; Figure S2; Tables 3 and Table S3); moreover, these primers had high amplification success. Of these 12 cpDNA markers, two primers (B13 and B36) revealed the highest mean intraspecific diversity (π) with the values of 0.0056 and 0.0051 across the 14 deciduous oaks. For the eight plastid regions having repetitive structures, two markers (R1 and R7) showed high DIPs with the mean values of 0.4290 and 0.5000, respectively (Table 3).

**Table 3:** **Information of plastid primers selected for phylogeography and species identification based on 14 deciduous oak species**.

**Primer ID**	**Amplification region**	**Forward (5′-3′)**	**Reverse (5′-3′)**	**Tm (°C)**	**Aligned length (bp)**	**Amplification success (%)**	**Mean diversity (π)**
B1^*^	*psb*A-*trn*K^UUU^	GACGGTTTTCAGTGCTGGTTATCC	TTTTCATCAATGGTCTGTCC	55	514	100	0.0016
B2^*^^#^	*mat*K-*trn*K^UUU^	TCTTACGATTTCTGCCCCTTCT	TTCTTAGCGGATCGGTTCAAAA	53	755	100	0.0008
B3^*^^#^	*rps*16	TTGGGATAGATGTAGATGAATAA	TCGGGGGTTGGGATGTAAATAGT	53	612	100	0.0012
B4^#^	*mat*K	ACCCAGTCCATCTGGAAATCTTGGTTC	CGTACAGTACTTTTGTGTTTACGAG	55	698	100	0.0002
B9^*^	*atp*I-*rps*2	ATAATGGCTGAACCTAATAAGATA	TCCCAGCAAATGATGACG	56	379	86	0.0006
B13^*^	*trn*D^GUC^-*trn*E^UUC^	CTTGACAGGGCGGTGCTCT	TAATGGGGACGGACTGTAAAT	54	491	100	0.0056
B14^*^	*psb*C-*lhb*A	ATTGATCGCGATTTTGAACCT	CCACGAATCTATTAATGCTGTATG	55	435	100	0.0018
B17^*^^#^	*ycf*3-*trn*S^GGA^	AAATCGCACCATCTCTGTAATAGG	CAAAACCGGGTGAATAGTGAGTC	58	884	100	0.0011
B29^*^	*rps*11-*rps*8	TATTCTACGCGCACTCTTACG	AACGGGTTTCTATTCTCACTCTC	55	661	100	0.0022
B30^*^	*rps*3-*rps*19	TGCAAACCAAAGAGAATGATGAC	TCCGAGGACACGCAAAAA	55	618	100	0.0012
B31^*^^#^	*ndh*F	TTCGGCCAATGCTCTTAT	TCCACCCCTTGCCTGTTTT	54	781	100	0.0007
B36^*^^#^	*rps*15-*ycf*1	ATCCCCTGTTTTCTTCTTTTTC	CAATAATGGTAGTCGTTTCAGTCT	53	493	100	0.0051
B37^*^^#^	*ycf*1^(5′)^	CGCGATAGGGTCCGTTCA	CTATTTAGGCAGAGTACCGTCACC	55	757	93	0.0008
B38^#^	*ycf*1	ATTCTGATGGTCCGGAAGGG	CCTTATCAGACTGAAACGACTAC	52	440	100	0.0006
B39^#^	*trn*H^GUG^-*psb*A	GTTATGCATGAACGTAATGCTC	CGCGCATGGTGGATTCACAATCC	62	332	100	0.0003
R1^*^	*rps*12-*trn*V^GAC^	CCAAATTGACGGGTTAGTGTGA	TTACCTCCGCGGAAAAGATGAT	55	415	100	0.429^a^
R7^*^	*rps*16-*trn*Q^UUG^	AAAGAAAATGATGGATGTAAGAAT	TAATGTAATGATAGTGAAATAGGA	54	483	100	0.5^a^

* Primers selected for phylogeographic analysis;

# Primers selected for species identification;

a *Values of Deletion-Insertion polymorphism (DIP)*.

For selection of candidate DNA barcodes, screening on amplicons revealed that nine primer pairs generated species-specific characters, which might show discriminating potential among the 14 deciduous oak species (Table 3). Overall, the selected cpDNA markers for phylogeographic and barcoding studies amplified 17 chloroplast regions, 12 were located in intergenic spacers, while four and one markers were anchored in codon and intron regions (Figure S3), respectively. In order to select powerful barcoding markers for all the Chinese oak species, the ABGD method was initially applied to sequences obtained from 14 deciduous oaks after PCR amplification of these nine primer candidates and all possible combinations. The ABGD results showed that most primer pair candidates demarcated one species groups and only primer pair B38 (*ycf*1) delineated three species groups, while ranges of intra- and interspecific genetic distances in each candidate barcode were overlapped based on the 14 deciduous oak species (Table S4). Despite this, five cpDNA markers (*psb*A-*trn*H, *mat*K-*trn*K, *ycf*3-*trn*S, *mat*K, and *ycf*1; Table 4) were chosen for our barcoding analyses because their combination revealed the highest species identification level among the 14 deciduous oaks tested.

**Table 4:** **Estimation of genetic information of seven barcode candidates in DNA barcoding analysis based on the 35 Chinese oak species**.

**Barcode ID**	**No. amplicons**	**Aligned length (bp)**	**No. variable sites**	**No. informative sites**	**No. indels**	**No. diagnostic indels**	**Maximum intraspecific distance (SE)**
*mat*K-*trn*K	205	694	37	33	2	1	0.0130 (0.0043)
*ycf*3-*trn*S	207	607	23	21	2	0	0.0099 (0.0038)
*psb*A-*trn*H	211	502	37	36	11	5	0.0139 (0.0052)
*mat*K	209	729	34	32	0	0	0.0123 (0.0038)
*ycf*1	216	448	29	29	0	0	0.0117 (0.0048)
ITS	201	411	96	86	2	1	0.0389 (0.0095)
SAP	173	355	52	39	0	0	0.0169 (0.0064)

### Barcode universality and sequence characteristics

The five plastid candidate barcodes and two nuclear genes showed high amplification success rates (100%) at the species level for the 35 Chinese oak species. At the individual level, these five cpDNA candidates as well as the ITS region had medium to high success rates (87–100%), while the SAP gene showed the lowest amplification rates in species from morphology-based Sections Quercus, Heterobalanus, Engleriana, and Echinolepides (Table 5).

**Table 5:** **Information of amplification success for the seven candidate barcodes used in this study**.

	**Barcode ID**	***Quercus***	***Cerris***	***Ilex***		
		**Quercus (%)**	**Aegilops (%)**	**Heterobalanus (%)**	**Engleriana (%)**	**Echinolepides (%)**
Species level	*mat*K-*trn*K	100	100	100	100	100
	*ycf*3-*trn*S	100	100	100	100	100
	*psb*A-*trn*H	100	100	100	100	100
	*mat*K	100	100	100	100	100
	*ycf*1	100	100	100	100	100
	ITS	100	100	100	100	100
	SAP	100	100	100	100	100
	Overall	100	100	100	100	100
Individual level	*mat*K-*trn*K	94	100	93	93	100
	*ycf*3-*trn*S	99	100	89	96	100
	*psb*A-*trn*H	100	100	94	93	100
	*mat*K	99	100	93	96	95
	*ycf*1	99	100	100	100	100
	ITS	96	94	87	93	95
	SAP	75	100	76	89	81
	Overall	95	99	90	94	96

A total of 1422 sequences of the 35 Chinese oak species were available for the DNA barcoding analyses. The aligned lengths of the seven barcodes ranged from 355 bp for the SAP gene to 729 bp of the *mat*K region. Among the seven barcodes, the aligned ITS region showed the highest number of variable sites, while the *ycf*3-*trn*S alignment had the lowest variable sites. The two nuclear genes exhibited more variable sites than the five plastid candidates. Of the seven tested barcodes, the *psb*A-*trn*H region had the highest number of indels (11) with five of them being diagnostic for some Chinese oak species. The *mat*K-*trn*K and *ycf*3-*trn*S sequences as well as the ITS region showed two indels in each alignment, but only one gap was species-specific for the *mat*K-*trn*K and ITS regions. No indels were found in the *mat*K, *ycf*1 and SAP alignments (Table 4).

### DNA barcoding gap assessment

**Figure 1:**
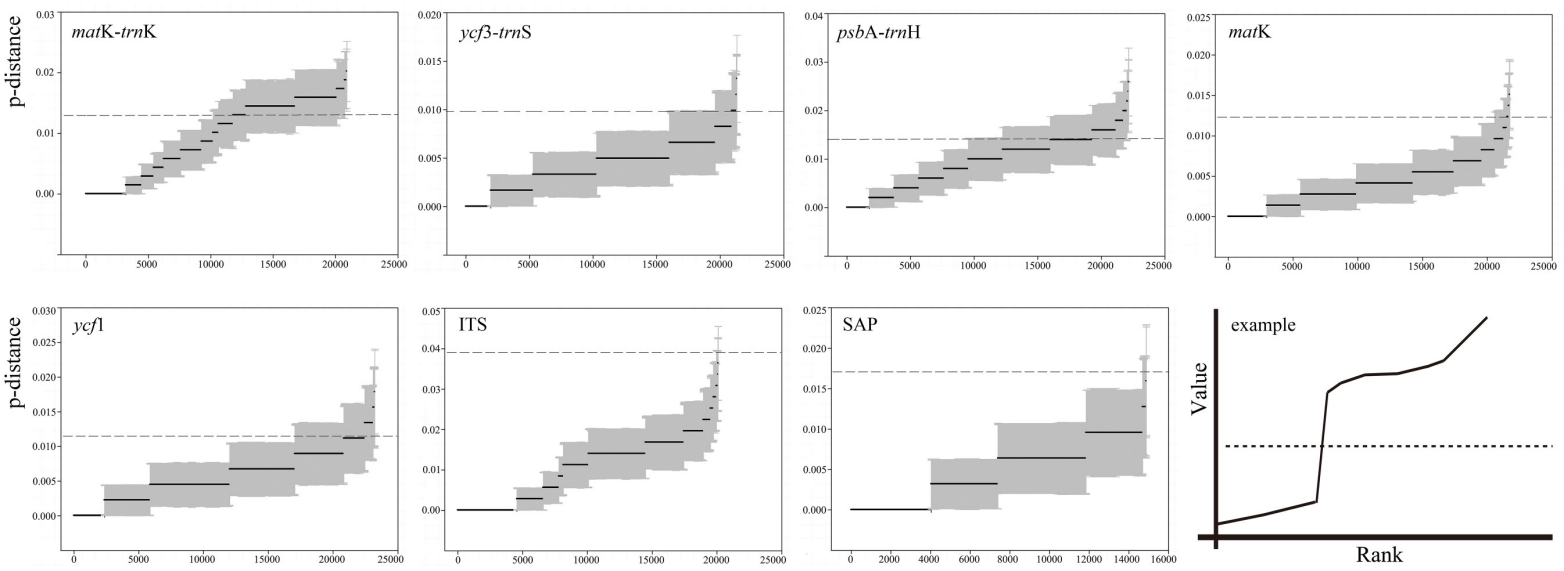
**Ranked values of genetic distances (p-distance) for the seven DNA barcode candidates among 35 Chinese oaks in ***Quercus*** subgenus ***Quercus***, and an example (Puillandre et al., 2012) indicating a clear barcoding gap**. X-axes represent the numbers of ranked genetic distances, Y-axes indicate the values of genetic distance. Black solid lines with gray areas show the estimated genetic distances with standard errors for each candidate. Dashed lines correspond to values of the maximum intraspecific divergence revealed for each candidate barcode.

Among the seven barcodes, the ITS region revealed the highest variation in the maximum intraspecific genetic distance, while *ycf*3-*trn*S showed the lowest maximum intraspecific variation (Table 4). No sudden increase was found around the vicinity of the maximum intraspecific distance for the ranked genetic distances, indicating no DNA barcoding gap among the 35 Chinese oak species for each barcode candidate (Figure 1). The differentiation tests between the minimum inter- and maximum intra-specific genetic distances revealed significantly higher values of the intraspecific variation than genetic variation at the interspecific level for *ycf*3-*trn*S, *mat*K, *ycf*1 and the two nuclear genes, while no differences between the intra- and inter-specific genetic distances were shown in *mat*K-*trn*K and *psb*A-*trn*H. Among species of each morphology-based section, the differentiation tests also showed significantly higher intraspecific variations than interspecific differentiation for Sections Quercus (corresponding to the phylogenetic Group *Quercus*), Aegilops (phylogenetic Group *Cerris*), Heterobalanus and Echinolepides, with the exception of Engleriana, for which the maximum intraspecific genetic distances were significantly lower than the minimum interspecific divergence (Table S5). However, when the differentiation tests were performed on the phylogenetic Group *Ilex* (including species of Sections Heterobalanus, Engleriana, and Echinolepides), both tests showed significantly higher intraspecific variations than interspecific differentiation among these evergreen oaks (*t* = 3.811, *P* <0.001; *W* = 32563.5, *P* <0.001).

### Comparison of species discrimination

**Figure 2:**
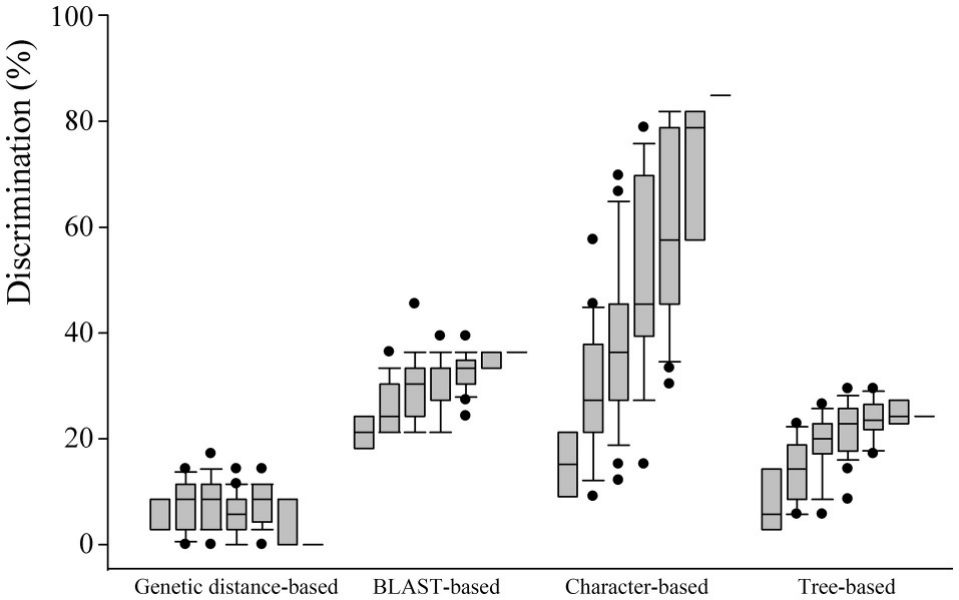
**Comparison of species resolution for four different barcoding methods (genetic distance-, BLAST-, character-, and tree-based methods) with single to seven barcode combinations based on the 35 Chinese oak species in ***Quercus*** subgenus ***Quercus*****. Box with error bars indicates 95% confidence intervals, and solid lines in the box show the mean values of species resolution. Black dots indicate outliers for specified barcode combination.

The four analysis methods afforded different rates of species discrimination for the 35 Chinese oak species in subg. *Quercus*. Overall, among the four different DNA barcoding methods, the character-based method showed the highest species discrimination rates with values from 30.30 to 84.85% for individual candidates to seven barcode combination. For a single barcode, the BLAST- and character-based methods provided the highest level of species identification (30.30%). The genetic distance-based method showed the lowest discrimination rates for the Chinese oak species for single barcodes and all their combinations, ranging from 0 to 17.14% (Figure 2; Tables S6, S7).

When comparing the discriminating potentials of each of the seven candidate loci and all of their combinations (Figure 2), ITS and the *psb*A-*trn*H region showed the highest species discrimination rate (both 30.30%) when the character-based method and the BLAST-based method were used, respectively. However, the ITS region identified no species group with the genetic distance-based method. For the character-based method, combinations with increasing numbers of barcoding markers tended to provide increasing rates of the species discrimination and showed the highest species discrimination potentials (84.85%) with the combination of *psb*A-*trn*H + *mat*K-*trn*K + *mat*K + *ycf*1 + ITS + SAP, as well as with all the seven barcode candidates. In contrast, for the three remaining methods, the species identification rates did not significantly increase when the barcode combinations included a larger number of candidate barcodes (Tables S6, S7). No species group was found for the combination of the seven barcoding markers when the genetic distance-based method was performed (Figure 2). The highest species identification rates of the genetic distance-, BLAST- and tree-based methods were 17.14% with a three-marker combination (*mat*K-*trn*K + *ycf*3-*trn*S + ITS), 45.45% using the combination of *mat*K + *ycf*1 + SAP, and 30.30% with six combined candidates of *psb*A-*trn*H + *mat*K-*trn*K + *ycf*3-*trn*S + *ycf*1 + ITS + SAP, respectively.

**Figure 3:**
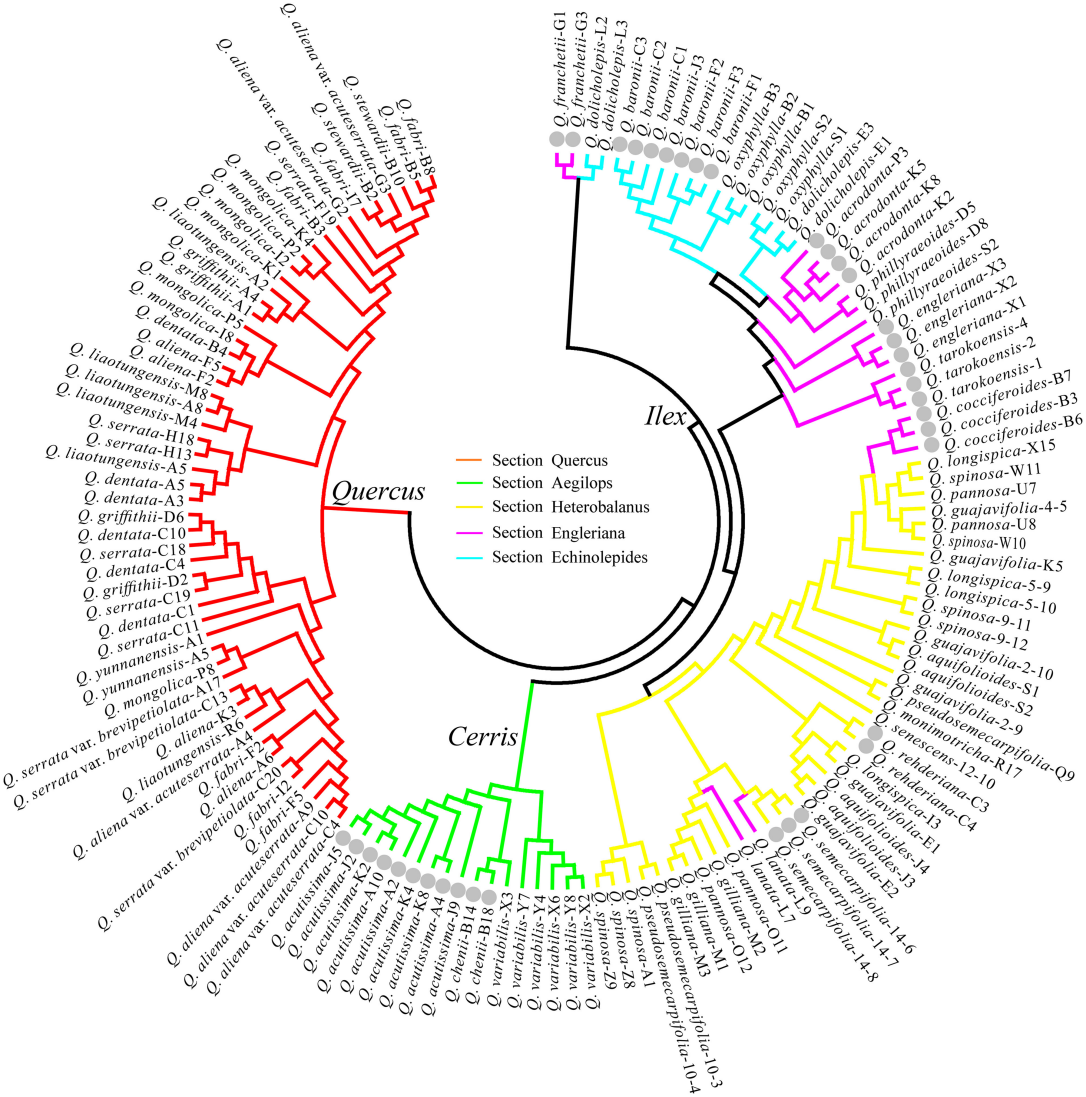
**NJ tree of the highest species identification rate with the combination of ***psb***A-***trn***H + ***mat***K-***trn***K + ***ycf***3-***trn***S + ***ycf***1 + ITS + SAP shows the phylogenetic implications for the Chinese oak species of ***Quercus*** subgenus ***Quercus*****. Gray dots highlight the individuals of identified oak species.

Along with the DNA barcoding aspects, the NJ trees generated were also used to test the five morphology-based sections Quercus, Aegilops, Heterobalanus, Engleriana, and Echinolepides, and the phylogenetic hypothesis of Groups *Quercus, Cerris* and *Ilex* for the Chinese oaks of subg. *Quercus*. The NJ tree with the highest species discrimination was shown in Figure 3. Ten of 33 Chinese oak species (*Quercus monimotricha* and *Quercus senescens* were not counted due only one sample of each in the analyses) were identified: two species in morphology-based Section Aegilops, two in Heterobalanus, five species in Engleriana, and one in Echinolepides. No monophyletic species clade was found in Section Quercus. At the infrageneric level, the five morphology-based sections were not well resolved in the NJ tree: all the white oaks of Section Quercus formed a distinct group; the three deciduous oak species from Aegilops also clustered together as a monophyletic group, and showed close relationship with the remaining evergreen oaks; while oaks of Sections Heterobalanus, Engleriana, and Echinolepides were mixed and formed multiple clades without clear section boundaries. In contrast, a previous phylogenetic classification for subg. *Quercus* was highly confirmed that individuals of the 35 Chinese oak species basically formed three major clades, corresponding to the phylogenetic Groups *Quercus, Cerris*, and *Ilex*. The NJ trees generated from four single barcodes (*psb*A-*trn*H, *mat*K-*trn*K, ITS, and SAP), the combinations of the five cpDNA barcodes and of two nuclear genes, as well as the combination of seven candidate loci showed similar phylogenetic patterns to the best species identification tree based on six barcodes (Figure 3), while for the NJ trees of the *ycf*3-*trn*S, *mat*K, and *ycf*1 regions, species of the three major groups were mixed without distinct boundaries except that the Group *Cerris* was identified by the *ycf*1 region (Figures S4–S13). The morphology-based Section Heterobalanus was only supported by a NJ tree using the combination of ITS and SAP (Figure S12), however, support for this monophyletic clade was low (Bootstrap probability = 58).

## Discussion

### Development of universal cpDNA markers for the Chinese oak species

The Chinese oak species in subg. *Quercus* show complex distribution patterns from the Qinghai-Tibetan Plateau to south and north China (Wang et al., 1985; Zhou, 1993), possibly reflecting complex population dynamics among species, as the past climatic and geographical impacts in different regions of China are considered heterogeneous (Harrison et al., 2001; Qiu et al., 2011). However, relatively few phylogeographic and phylogenetic studies have been performed for the Chinese oak species, probably due to the ambiguous species boundaries and low universality and genetic variation of current molecular markers. To overcome these limitations, the chloroplast genomes of *Q. rubra* and *C. mollissima* were compared to develop universal cpDNA markers. Two reasons were considered for this comparison: (1) among the complete and available Fagaceae genomes, these two species are closely related taxa. Further, previous comparative genomic analysis, as well as phylogenetic investigations, have shown that oaks are more closely related to chestnuts than to other taxa in Fagaceae (Manos and Steele, 1997; Kremer et al., 2012); (2) our recent comparative work on the chloroplast genomes of five Chinese oak species indicates highly conserved identity of sequences across these oak genomes, suggesting limited genetic variation at low taxonomic levels (Yang Y. C. et al., 2016). The universal plastid markers developed in this study are envisioned to provide useful tools for phylogenetic, biodiversity and population studies of oak species, as well as for other genera in the Fagaceae.

In this study, 14 deciduous oak species with multiple samples per species were used to test the designed cpDNA markers rather than screening them with pairs of species (Shaw et al., 2007). Most of the designed cpDNA markers can be amplified across the 14 oaks, suggesting superior universality of these primer pairs for the Chinese oaks (Tables 3 and Table S2). After screening on the 14 deciduous oaks, we suggested 12 plastid regions generating high intraspecific diversity that could be useful for phylogeographic and population genetic studies. As expected, most of the 12 chloroplast markers amplify intergenic spacers (Figure S3), supporting the intergenic regions accumulate more intraspecific polymorphism than the codon regions in chloroplast genome (Shaw et al., 2007; Yang Y. C. et al., 2016). Additionally, two pairs of primers (R1 and R7) exhibiting high DIPs by introducing indels in individual sequences may provide additional genetic information for evolutionary processes of oak species having slow evolutionary rates (Table 3). For selection of candidate cpDNA barcodes, nine primer pairs having discriminatory potentials were chosen based on the results of the 14 deciduous oak species examined. Despite the limited species identification of each candidate (Table S4), five of them (Table 4) were chosen for testing DNA barcoding in 35 Chinese oak species. Evaluation of the five plastid candidates and two additional nuclear genes tested on the 35 Chinese oak species are discussed below.

### Evaluation of candidate DNA barcodes for the Chinese oaks

An efficient DNA barcode is expected to have highly universal primer pair as well as high interspecific variation, and to provide steady species-specific characters that allow species identity (Erickson et al., 2008; Hollingsworth et al., 2011; Naciri et al., 2012). The five candidate cpDNA barcodes selected in this study, as well as the two newly designed nuclear genes (ITS and SAP), showed relatively high universal coverage of the 35 Chinese oak species at the species and individual levels (Table 5), while their identification powers were variable across the Chinese oaks under different barcoding methods (Tables S6, S7).

Of the seven candidate barcodes, ITS and *psb*A-*trn*H provided the highest species resolution (both 30.30%) using the character- and the BLAST-based method, respectively. The ITS region was proposed as one of the core and powerful barcodes for seed plants (China Plant BOL Group, 2011), and its utility has also been supported in several barcoding studies (Liu et al., 2011, 2014; Muellner et al., 2011; Pang et al., 2011; Zhang et al., 2012; Chen et al., 2014; Zhang J. Q. et al., 2015). In a barcoding study of Euro-Mediterranean oaks, ITS2 increased the species resolution when combined with two plastid barcoding loci (Simeone et al., 2013); however, by itself, the ITS region showed no species identification when the genetic distance method was applied. Similarly, a barcoding study of *Rhododendron*, using ITS, showed low species resolution; this aspect could be attributed to recent radiative speciation, hybridization and introgression (Yan et al., 2015). The contrast performance of ITS in species identification of the 35 Chinese oaks between the character- and genetic distance-based analyses can be induced by technical difference of methods between these two barcoding approaches, as the rapid evolution rate of ITS could accumulate high intra- and inter-specific mutations, concealing interspecific genetic divergence among species; conversely, the character-based method synthesizes a set of diagnostic positions to distinguish species but ignores uninformative sites in the sequences (Bertolazzi et al., 2009), which may reduce the impact of intraspecific genetic variations. Obviously, with the character-based method, species identification rates of the Chinese oaks were increased when the ITS region was combined with other candidate barcodes (Table S7), supporting the utility of this region as an efficient barcode in plants.

As a single-copy gene, the SAP region was used to test the potential of low-copy nuclear loci as suitable barcodes for *Quercus*. Although there were amplification failures on some samples, the SAP gene showed 100% amplification success at the species level (Table 5). However, the SAP gene showed relatively low rate of species identification among the seven candidate barcodes (i.e., 2.86% in the genetic distance-based method and 18.18% using the BLAST-based method). Most combinations between the SAP gene and the remaining candidates provided inconspicuous promotions on the species discrimination rate except when the ITS region was joined (Table S7). The limited nucleotide variation and parsimonious informative sites of the functional SAP gene (Table 4), which yield low interspecific differentiations among closely related oak species, account for its poor species identification rate. Interestingly, despite the frustrating rate of species identification for the SAP gene, individuals of a widespread deciduous oak, *Q. acutissima*, seem to be well differentiated from two closely related species (*Q. variabilis* + *Quercus chenii*) (Figures 3, Figures S10, S12). One stable transition (in *Q. acutissima*) was found between *Q. acutissima* and *Q. variabilis* + *Q. chenii* in the alignment of SAP, which may induce potential differentiation of the stress-associated protein function encoded by this gene (Vij and Tyagi, 2006), and foster ecological differentiation among sympatric oaks (Cavender-Bares et al., 2015), particularly between the two widespread species *Q. acutissima* and *Q. variabilis*. However, given that only a few samples of the three closely related oaks were included in this study, the assumption that the SAP gene is likely a “speciation gene” (Wu, 2001) bears further verification with the study of a larger number of individuals and a functional proteomics approach.

Of the five plastid candidate barcodes, the *psb*A-*trn*H region showed the highest species resolution (30.30%) using the BLAST-based method. Due to its high level of sequence divergence and species identification rate, the non-coding *psb*A-*trn*H region has been suggested as a robust DNA barcode for plants (Kress et al., 2005; Hollingsworth et al., 2011; Taylor and Harris, 2012), and has been supported in several studies (Liu et al., 2014; Yan et al., 2015; Zhang J. Q. et al., 2015). In this study, the *psb*A-*trn*H region showed the highest number of informative sites, as well as diagnostic indels compared to the rest of plastid barcode candidates (Table 4). Additionally, the proposed problematic ambiguous inversions within *psb*A-*trn*H region encountered in some Euro-Mediterranean oaks (Simeone et al., 2013) was not found among the Chinese oak species, suggesting this region as a suitable core DNA barcode for *Quercus*. The *ycf*1 region is functional and exists in all Chinese oak species tested in this study, and showed the highest species resolution (14.29%) when the tree-based method was considered. This cpDNA region also provided considerable species discrimination rates compared to the *psb*A-*trn*H region using the other three methods, supporting that this locus should be included as a marker for barcoding analyses at low taxonomic levels (Neubig et al., 2009; Dong et al., 2012, 2015). However, given the high variation of the *ycf*1 gene that was verified in some angiosperm taxa (Dong et al., 2015), universality of the newly designed *ycf*1 region in this study bears further verification in other seed plants. The *mat*K-*trn*K region showed higher species resolution rates than *mat*K among most of the four barcoding methods, probably due to higher sequence variation in the intergenic region of *mat*K-*trn*K compared to the coding region of *mat*K. Thus, although these two regions are adjacent to each other (Figure S3), it seems better to use the *mat*K-*trn*K region for barcoding *Quercus* than *mat*K. The *ycf*3-*trn*S had the lowest sequence variation (Table 4) and showed relatively low species differentiation among the five plastid candidates. Combinations of the *ycf*3-*trn*S region with other candidate barcodes provided very little or no improvement of species resolution for the Chinese oak species, suggesting that this region could be omitted from barcoding studies in *Quercus*.

Overall, the seven barcode candidates show low rates of species identification among the 35 Chinese oaks when analyzed individually. Nevertheless, the combinations of DNA barcodes improved species discrimination, and provided varying species resolution with different barcoding methods (Figure 2; Table S6). The highest species discrimination rate (84.85%) was found using the combination of *psb*A-*trn*H + *mat*K-*trn*K + *mat*K + *ycf*1 + ITS + SAP with the character-based method, which is the same as the result obtained with the combination of all seven candidates (Tables S6, S7), and possibly acts as the best choice for barcoding the Chinese oaks. However, given the different performances among the four barcoding methods tested, choosing a suitable barcoding method is also crucial for barcoding analyses (Yan et al., 2015).

### Comparison of different analytical methods for barcoding the Chinese oaks

Among the four analytical methods, the character-based method provided the highest species resolution when individual barcode candidate and all of their combinations were used. Conversely, the genetic distance-based method showed the lowest rates of species identification, particularly with the combination of all the seven candidates, which assigned no species groups to the 35 Chinese oaks. The BLAST-based method exhibited higher species discrimination than the tree-based method, but was lower than the character-based approach (Figure 2; Table S6). The different performances of the four methods used in this study possibly reflect the variant analytical theories of the approaches implemented: the character-based method identifies potential distinctive nucleotide positions from DNA barcode sequences (the reference database) and assigns species using logic formulas based on the species-specific (diagnostic) codes (Table S8), which is suggested to be efficient and precise (van Velzen et al., 2012; Weitschek et al., 2013). Our results strongly support the highly discriminate capacity (84.85%) of this method, even when tested in *Quercus*, which is widely known as a taxonomically problematic group (Dumolin-Lapegue et al., 1999; Aldrich and Cavender-Bares, 2011). In addition, the highest species identification rate obtained with this approach is comparable to the average rate of successful species discrimination (70%) found in land plants (Ran et al., 2010; Hollingsworth et al., 2011), and also shows considerable ability of species identification compared with some previous researches in species-rich and recently divergent plant lineages using multiple DNA barcodes (Ran et al., 2010; Zhang et al., 2012; Simeone et al., 2013; Liu et al., 2014; Zhang J. Q. et al., 2015). However, caution should be taken as it is hard to provide a reliable/idealistic DNA barcoding reference containing specimens with *a priori* known species when not all the individuals of a species are tested against the putative barcode. Besides, the evolutionary processes of a species, such as local adaptation and hybridization, may obscure species identification by introducing negative mutations to the diagnostic sites in short DNA barcodes. Therefore, rather than indicating the species identification rate among the Chinese oaks, the character-based method may suggest possibilities of the barcode candidates that act as DNA barcodes to the oak species with those samples involved in this study, and bear further verification with additional individuals from unsampled areas. The BLAST-based method calculates the similarity of sequences with the assumption that conspecific individuals will be more similar to each other than to any other species (van Velzen et al., 2012). This method also yields higher identification rates than the distance- and tree-based methods as documented in several barcoding studies (Hassel et al., 2013; Chen et al., 2014; Yan et al., 2015). The tree-based approach is a bottom-up clustering algorithm that forms a single tree based on genetic distance matrix; however, the result could be affected by the distance algorithm used for tree construction (Yan et al., 2015), and by the incomplete lineage sorting effects among recently diverged species (Simeone et al., 2013). The (genetic distance-based) ABGD analysis has been proposed as a method sensitive to the presence of recent speciation events; in this method, species delimitation is based on the partition of intra- and inter-specific genetic distances of species datasets (Puillandre et al., 2012). However, in our study, application of this method seems impractical for barcoding the Chinese oak species given that the intra- and inter-specific genetic distances of the seven barcode candidates largely overlap (Figure 1; Table S5). Moreover, as it was concluded in a DNA barcoding test on Euro-Mediterranean oaks by Simeone et al. (2013), an increased sampling may lead to a continuum of overlapping genetic distances among closely related species, thus discouraging the successful species identification based on the genetic distance approach. The lack of a barcode gap among congeners was also observed for the Italian oak species, a probable consequence of low variation rate, frequent interspecific hybridization, and the complex incidence of biogeography of some sympatric species (Piredda et al., 2011).

### Phylogenetic implications for the Chinese oak species

Despite the overall poor performance of DNA barcoding for the Chinese oak species, the (NJ) tree-based results provided considerable insights into phylogenetic relationships of the Chinese oaks in subg. *Quercus*. The NJ tree with the highest species discrimination (Figure 3) largely confirms the traditionally recognized *Quercus* infrageneric Groups *Quercus, Cerris*, and *Ilex* (Denk and Grimm, 2009, 2010; Denk and Tekleva, 2014; Hubert et al., 2014). This infrageneric framework is also supported by the NJ trees generated with two cpDNA barcode candidates (*psb*A-*trn*H and *mat*K-*trn*K) (Figures S4, S5) and two nuclear genes (ITS and SAP) (Figures S9, S10) respectively. While for the three remaining cpDNA barcodes, the NJ trees indicated unclear boundaries among the three major groups of subg. *Quercus* (Figures S6–S8), probably due to a lower mutation rate and less genetic information of these cpDNA markers obtained among the Chinese oaks when compared with that of the intergenic *psb*A-*trn*H and *mat*K-*trn*K regions and the two nuclear genes (Table 4). In contrast, the five morphology-based sections (Zhou et al., 1995; Pu et al., 2002; Peng et al., 2007) are controversial. Oak species of the Group *Quercus* (morphology-based Section Quercus) are clearly distinct from the clade comprising oaks of Groups *Cerris* and *Ilex*, strongly supporting a scenario of two possible originations (the New World and Old World Oaks) of *Quercus* that was previously identified with phylogenetic classification (Oh and Manos, 2008; Denk and Grimm, 2009, 2010; Denk and Tekleva, 2014; Hubert et al., 2014). In China, the three deciduous East Asian species of Group *Cerris* (morphology-based Section Aegilops) are strongly supported forming a monophyletic clade in contrast to the shared “*Cerris*-*Ilex*” haplotype scenario found in some Eurasian oaks (Denk and Grimm, 2010; Hubert et al., 2014; Simeone et al., 2016). Pollen morphology of the three cerriod oaks in East Asia (*Q. acutissima, Q. variabilis*, and *Q. chenii*) is different from the evergreen oaks of Group *Ilex* in China (Cao and Zhou, 2002), thus suggesting possible reproductive isolation between the cerriod and ilicoid oaks in China. However, although the hybrid formations are considered to be highly frequent among closely related species within a section/group, hybridization and gene flow between oak species from Groups *Cerris* and *Ilex* were found in Europe, such as hybridization between *Quercus suber* and *Q. ilex*, which may reflect enigmatic species evolutionary history of the two major Old World clades (Groups *Cerris* and *Ilex*) linked with complex incidence of geographical effects during different periods in Europe (Aldrich and Cavender-Bares, 2011; Hubert et al., 2014; Simeone et al., 2016). Within Group *Ilex*, species of the morphological Sections Heterbalanus, Engleriana, and Echinolepides are mixed and form multiple clades without clear boundaries in the NJ trees (i.e., Figures 3 and Figure S13), albeit the oaks among the three morphology-based sections show distinct morphological differences in foliage and acorn cupule patterns (Pu et al., 2002; Peng et al., 2007). Previous phylogenetic studies on Eurasian oaks involving a fraction of these evergreen Asian species indicated similar patterns, highlighting the incongruence between morphology and phylogenetic relationships in *Quercus* (Denk and Grimm, 2010; Simeone et al., 2016). Prior morphological and phylogenetic studies have provided evidences that Section Heterobalanus is an expanded/natural group within Group *Ilex* (Zhou et al., 1995; Manos et al., 2001; Deng et al., 2017); however, a morphologically distinct species (*Quercus engleriana*) was found to be paraphyletic within the Heterobalanus clade in that phylogeny (Manos et al., 2001), and our phylogenetic implications of NJ trees also rejected the monophyly of Section Heterobalanus (Figure 3 and Figure S13) except for a NJ tree using the combination of ITS and SAP (Figure S12), which slightly supported the Heterobalanus subclade (BP = 58) within Group *Ilex*. The sclerophyllous alpine oaks in Section Heterobalanus are largely sympatric and mainly concentrated in the Hengduan Mountains and adjacent alpine regions in east of the Himalayas, where they chiefly occur above 2000 m (Yang et al., 2009). Specific morphological characteristics of the alpine oak species in Section Heterobalanus, such as dense hairs, thick cuticles and lignified epidermal cell walls, are believed to be initiated by the cold and dry conditions of the Hengduan Mountains since the Miocene to Pliocene (Zhou et al., 2003), suggesting potential ecological niche shift of these alpine oaks to the intense orographic and climatic turbulence around the Qinghai-Tibetan Plateau. The hypothesis of the subclade Heterobalanus within Group *Ilex* based on two combined nuclear genes in this study also indicates that nuclear genes may provide more efficient phylogenetic signals than chloroplast genes having low mutation rate in recently divergent lineages. However, given the limited molecular markers and phylogenetic methodology used in the DNA barcoding test, the monophyly of Section Heterobalanus in Group *Ilex* still needs further verification with more functional nuclear genes. In summary, the lack of clear boundaries of the three evergreen morphological sections in Group *Ilex* can be explained by incomplete lineage sorting and/or gene introgression between closely related species (Manos et al., 2001; Denk and Grimm, 2010), as well as by limited genetic information revealed in current phylogeny, which requires further phylogenetic evidences using more comprehensive genomic coverage.

At the species level, the NJ tree with the highest discrimination rate identified 10 Chinese oak species in subg. *Quercus*, most of which are evergreen oaks in the *Ilex* group, while no species resolution was found among Chinese white oaks (Group *Quercus*) (Figures 3 and Figure S13). The identified evergreen species, for example *Quercus franchetii, Quercus acrodonta, Quercus cocciferoides* and *Q. engleriana*, are highly diverse in morphology and karyotype (Cao and Zhou, 2000), and evolved early according to fossil data (Zhou, 1993). Additionally, these oaks are endemic and allopatric, suggesting long-time isolation due geographical barriers among these species, which may have enhanced genetic and morphological differentiation during species evolution. This aspect is also found among the European oak species of Group *Ilex*, which showed conspicuous plastid diversity, reflecting complex geographical history and growing geographic isolation among members of this clade (Simeone et al., 2016). In contrast, some white oak species of Group *Quercus*, such as *Q. aliena, Quercus fabri*, and *Quercus dentata*, are widespread tree species across south China (Wang et al., 1985). Adaptive divergence to different ecological niches due extensive distribution could explain the species identification failure for this group. Additionally, fossil evidences and previous researches suggested recently radiative diversification of the closely related species involved in Group *Quercus* (Zhou, 1992; Hubert et al., 2014; Yang J. et al., 2016), which possibly indicates incomplete lineage sorting and shared ancestral polymorphism among these deciduous oak species. Moreover, according to the *flora of China*, flowering time of these white oaks highly overlaps from March to May, suggesting that frequent interspecific hybridization may occur among recently diverged species, thus eroding species coherence in this group. The latter explanation, as well as introgression, incomplete lineage sorting and reticulation (Simeone et al., 2013), have also been suggested as potential processes that complicate species identification of the morphology-based Section Heterobalanus (if it exists), which comprises 8–11 alpine sclerophyllous oaks that mainly happen in the Hengduan Mountains (Yang et al., 2009) and show identical florescence covering May and June.

Overall, the NJ results of the DNA barcoding analyses in this study highlighted the phylogenetic relationship and evolutionary significance of the Chinese oak species in subg. *Quercus*, and provided additional taxonomic patterns for the diverse oak lineage. However, considering the few DNA barcode candidates and a simple genetic distance method (p-distance) used for constructing NJ trees in our DNA barcoding analyses without regarding any evolutionary model and compatibility among these barcode candidates, the phylogenetic implications obtained from the NJ results in this study may be insufficient compared with that of a specific phylogenetic research, which warrant particular investigations using comprehensive samplings and analytical methods, as well as with more efficient genetic information covering the genomic level (Zhang et al., 2012; Simeone et al., 2013).

## Conclusions

In this study, a comparative analysis on two chloroplast genomes (*Q. rubra* and *C. mollissima*) in Fagaceae was adopted to explore potential universal cpDNA markers for *Quercus*. After laborious screening on a subset of 14 deciduous Chinese oaks, 14 primer pairs revealing high intraspecific genetic diversity in most of the species dataset were selected for phylogeographic studies, while nine plastid regions exhibiting species discriminate potentials were retained for barcoding *Quercus*. Five of the nine plastid candidate barcodes, with additions of the proposed ITS region and a single-copy nuclear gene (SAP) were then used to test their discriminatory power employing four different barcoding methods for 35 Chinese oak species. Among the seven barcode candidates, the ITS and *psb*A-*trn*H regions provided the highest species resolution (30.30%) using the character- and BLAST-based method, respectively, and were recommended as core DNA barcodes for *Quercus*. The highest species discrimination rate (84.85%) was found using the combination of *psb*A-*trn*H + *mat*K-*trn*K + *mat*K + *ycf*1 + ITS + SAP with the character-based method, which seems to be the best choice for barcoding the Chinese oaks. The character-based method showed the best potential for species identification among the four barcoding methods, which is supported as an efficient tool for barcoding lineages having recently diverged species. The DNA barcoding results also provided phylogenetic implications for the framework of the Chinese oak species which has yet been assessed, supporting the phylogenetic classification of three major groups *Quercus, Cerris* and *Ilex*; however, the previously recognized five morphology-based sections proposed for the Chinese oaks are not well recovered. Some factors, such as low evolutionary rate of the plastid genome, interspecific hybridization and introgression, and incomplete lineage sorting, are likely to affect the phylogenetic signal in Chinese oaks and lead to intractable species identification based on different barcoding methods.

## Author contributions

JY and GZ designed the research. JY, XC and HL performed the experiments. JY, XC, HL, and HZ contributed materials and data analysis. JY and LV wrote the paper. LV, ZL and GZ revised the paper. All authors read and approved the final manuscript.

### Conflict of interest statement

The authors declare that the research was conducted in the absence of any commercial or financial relationships that could be construed as a potential conflict of interest.

## Supplementary material

Figure S1 **Annotation of the complete chloroplast genome of *Quercus rubra***. Inner circle indicates inferred structures of a complete chloroplast genome. Black boxes on the outer circle represent the selected regions for primer design.

Figure S2 **Estimation of mean intraspecific diversity (π) for 12 selected cpDNA markers (in Table 3) based on a subset of 14 Chinese oak species from morphology-based Sections Quercus and Aegilops**. Box plots represent 95% confidence intervals and solid lines in the box plots indicate mean diversity values. Black dots indicate outlying values.

Figure S3 **Priming sites of the 17 selected cpDNA markers (Table 3) in chloroplast genome of *Quercus rubra***. White boxes indicate relative coding positions of chloroplast genes for primer design and gene names are displayed above. Black and gray lines indicate intergenic spacer and intron region, respectively. Numbers above the lines show the intergenic spacer and intron length (bp). Marker IDs are displayed in bold.

Figure S4 **NJ tree of the single barcode candidate *psb*A-*trn*H region for the Chinese oak species in *Quercus* subgenus *Quercus***.

Figure S5 **NJ tree of the single barcode candidate *mat*K-*trn*K region for the Chinese oak species in *Quercus* subgenus *Quercus***.

Figure S6 **NJ tree of the single barcode candidate *ycf*3-*trn*S region for the Chinese oak species in *Quercus* subgenus *Quercus***.

Figure S7 **NJ tree of the single barcode candidate *mat*K region for the Chinese oak species in *Quercus* subgenus *Quercus***.

Figure S8 **NJ tree of the single barcode candidate *ycf*1 region for the Chinese oak species in *Quercus* subgenus *Quercus***.

Figure S9 **NJ tree of the single barcode ITS region for the Chinese oak species in *Quercus* subgenus *Quercus***.

Figure S10 **NJ tree of the single-copy nuclear gene (SAP) for the Chinese oak species in *Quercus* subgenus *Quercus* highlights the species discrimination of *Quercus acutissima* from *Quercus variabilis* + *Quercus chenii* in Group *Cerris***.

Figure S11 **NJ tree based on the combination of five cpDNA barcode candidates for the Chinese oak species in *Quercus* subgenus *Quercus***.

Figure S12 **NJ tree based on the combination of two nuclear genes for the Chinese oak species in *Quercus* subgenus *Quercus* highlights the species discrimination of *Quercus acutissima* from *Quercus variabilis* + *Quercus chenii* in Group *Cerris* and shows the monophyletic subclade of Section Heterobalanus in Group *Ilex***.

Figure S13 **NJ tree of the combination with all seven candidate barcodes shows the phylogenetic implications for the Chinese oak species in *Quercus* subgenus *Quercus***. Gray dots highlight the individuals of identified oak species.

Table S1 **Information of voucher specimens of the 35 Chinese oak species in *Quercus* subgenus *Quercus***.

Table S2 **Information of 49 designed cpDNA markers based on the comparison of two chloroplast genomes of *Quercus rubra* and *Castanea mollissima***.

Table S3 **Estimation of intraspecific genetic diversity (π) for 14 deciduous oak species of morphology-based Sections Quercus and Aegilops based on the designed cpDNA markers**.

Table S4 **Estimation of genetic information and Automatic Barcode Gap Discovery (ABGD) results for nine candidate barcodes based on 14 Chinese oak species**.

Table S5 **Differentiation tests between the minimum interspecific and maximum intraspecific genetic distance (p-distance) for each barcode candidate and section**.

Table S6 **Estimation of the highest species discrimination rates for four barcoding methods based on single and all possible barcode combinations**.

Table S7 **Species resolution of seven single barcodes and all their possible combinations for the Chinese oaks based on four barcoding methods**.

Table S8 **The minimum cost logic formula (diagnostic nucleotide) of the highest species identification using six barcode candidates (psbA-trnH + matK-trnK + matK + ycf1 + ITS + SAP) for the Chinese oaks**.

Note S1 **Detailed methods for data filtering of the ITS sequence**.
