# Supplementary figures and images for: Development of Chloroplast and Nuclear DNA Markers for Chinese Oaks (Quercus Subgenus Quercus) and Assessment of Their Utility as DNA Barcodes
Source: Front Plant Sci. 2017 May 19;8:816. doi: 10.3389/fpls.2017.00816 (PMC5437370; doi:10.3389/fpls.2017.00816)

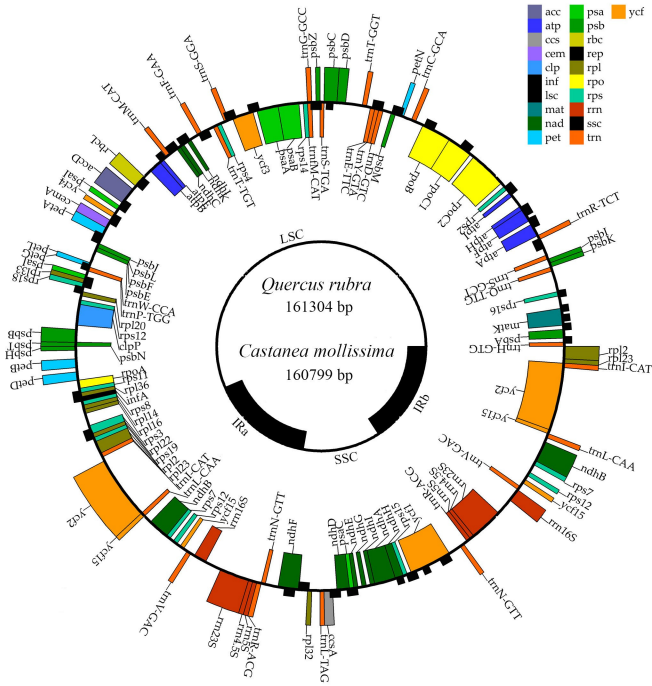

Supplement: Figure S1 — Annotation of the complete chloroplast genome of Quercus rubra. Inner circle indicates inferred structures of a complete chloroplast genome. Black boxes on the outer circle represent the selected regions for primer design. [file Image1.PDF]

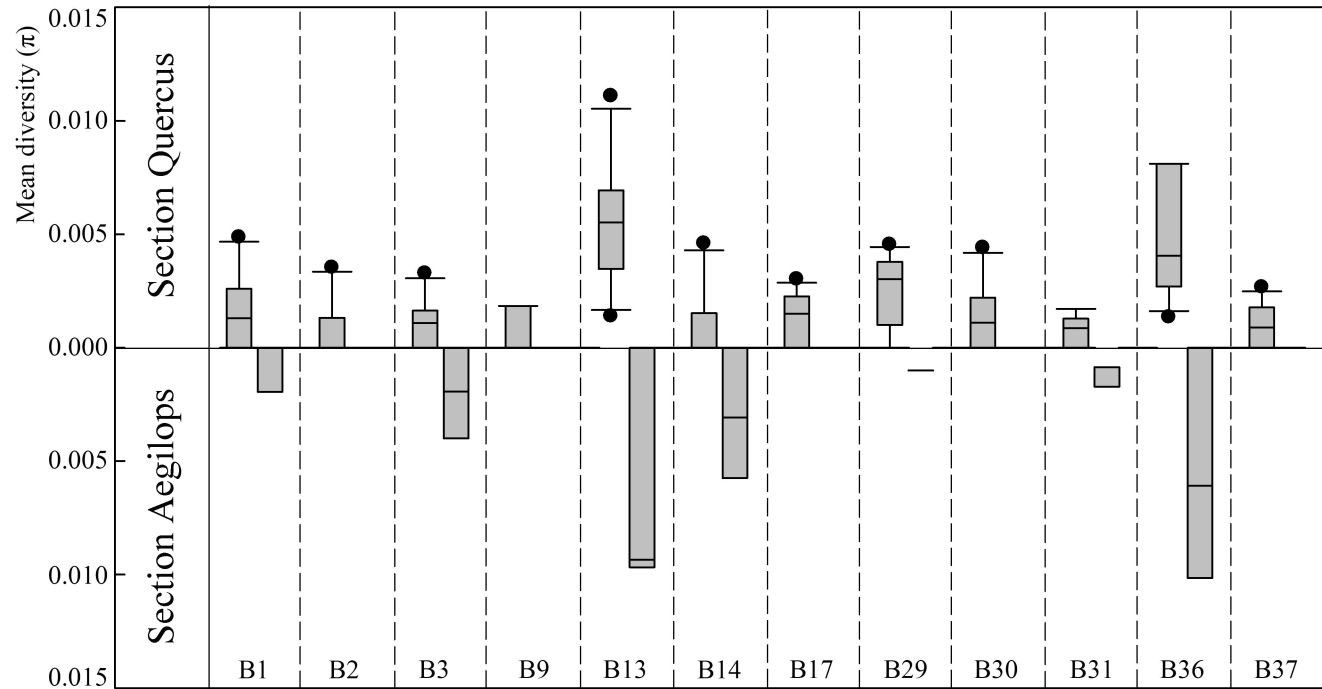

Supplement: Figure S2 — Estimation of mean intraspecific diversity (π) for 12 selected cpDNA markers (in Table 3) based on a subset of 14 Chinese oak species from morphology-based Sections Quercus and Aegilops. Box plots represent 95% confidence intervals and solid lines in the box plots indicate mean diversity values. Black dots indicate outlying values. [file Image2.PDF]

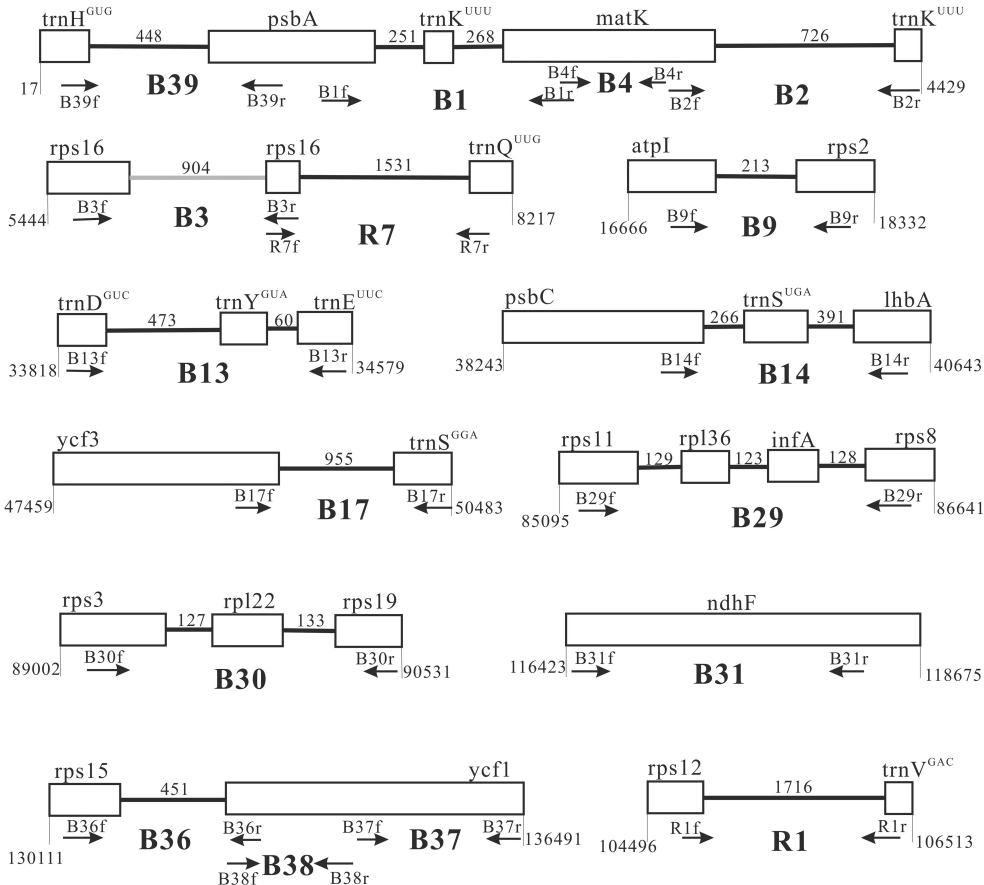

Supplement: Figure S3 — Priming sites of the 17 selected cpDNA markers (Table 3) in chloroplast genome of Quercus rubra. White boxes indicate relative coding positions of chloroplast genes for primer design and gene names are displayed above. Black and gray lines indicate intergenic spacer and intron region, respectively. Numbers above the lines show the intergenic spacer and intron length (bp). Marker IDs are displayed in bold. [file Image3.PDF]

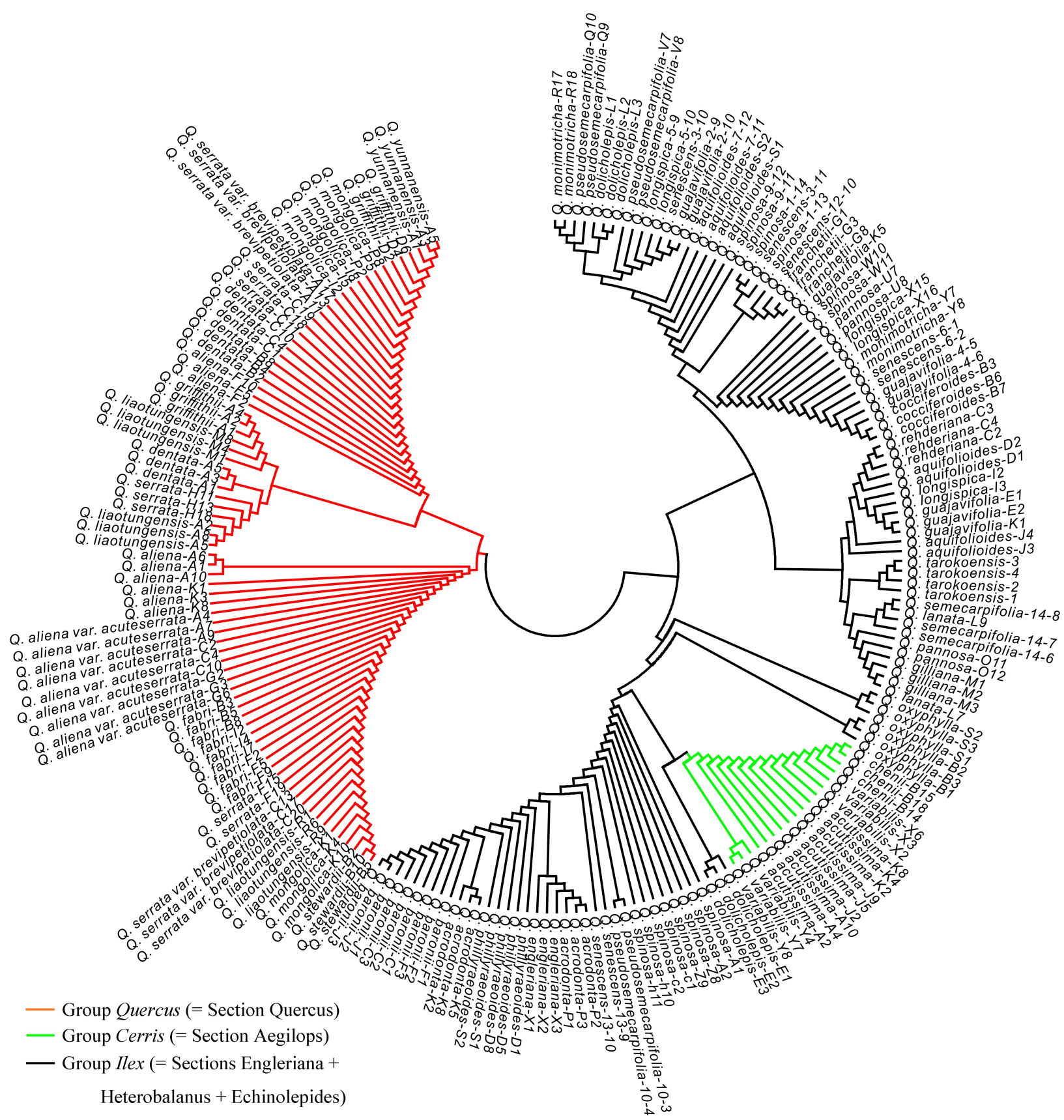

Supplement: Figure S4 — NJ tree of the single barcode candidate psbA-trnH region for the Chinese oak species in Quercus subgenus Quercus. [file Image4.PDF]

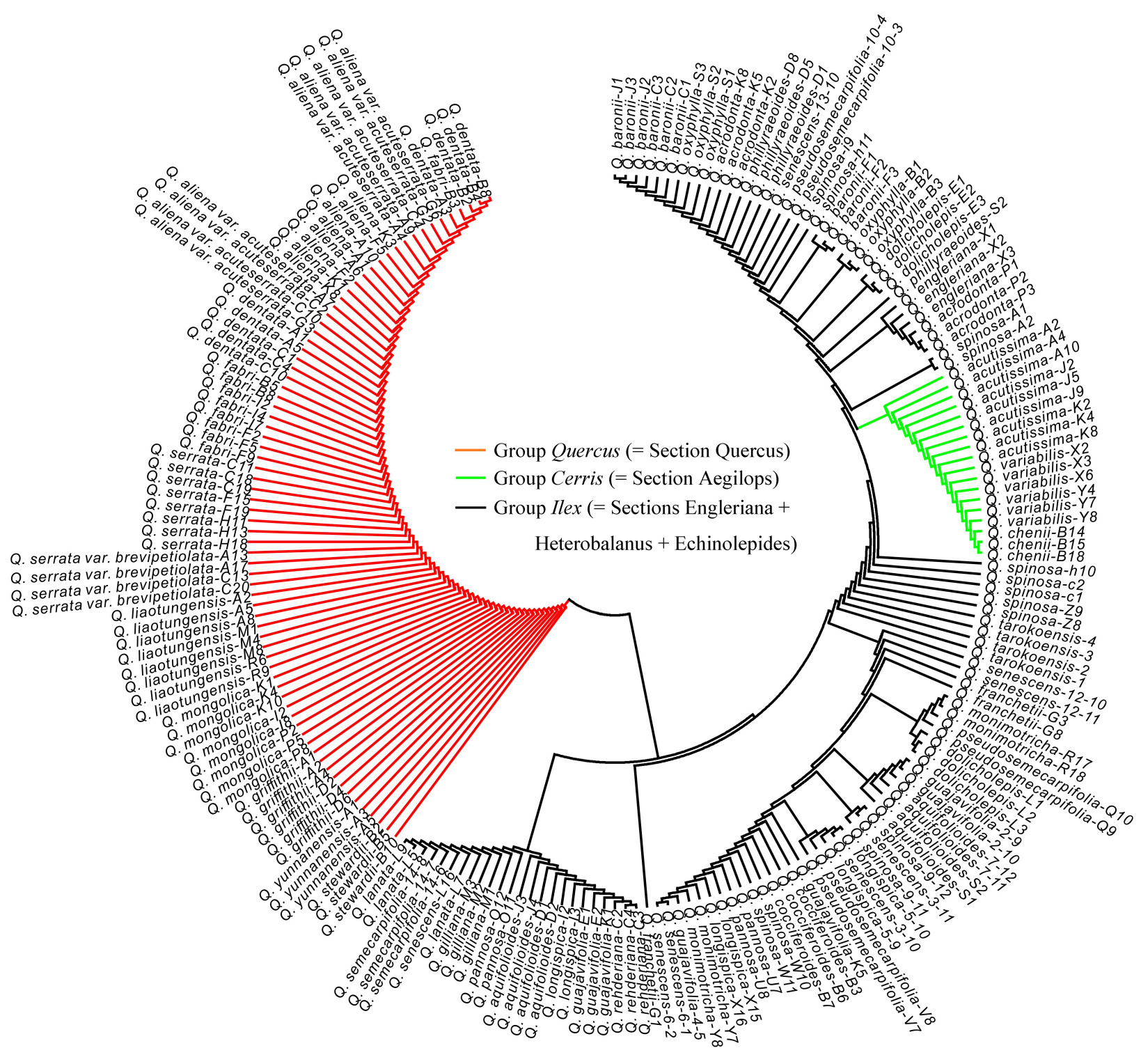

Supplement: Figure S5 — NJ tree of the single barcode candidate matK-trnK region for the Chinese oak species in Quercus subgenus Quercus. [file Image5.PDF]

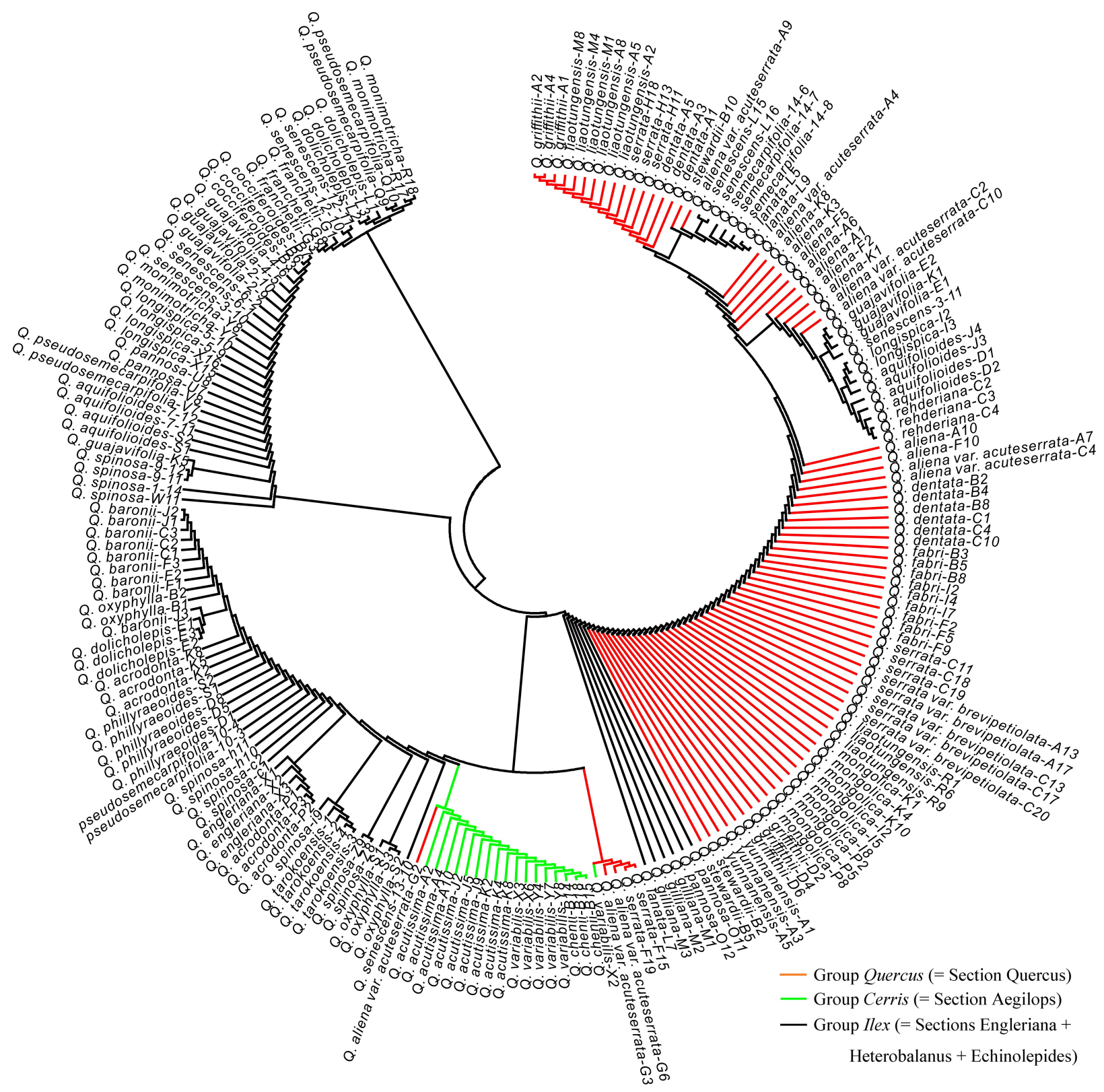

Supplement: Figure S7 — NJ tree of the single barcode candidate matK region for the Chinese oak species in Quercus subgenus Quercus. [file Image7.PDF]

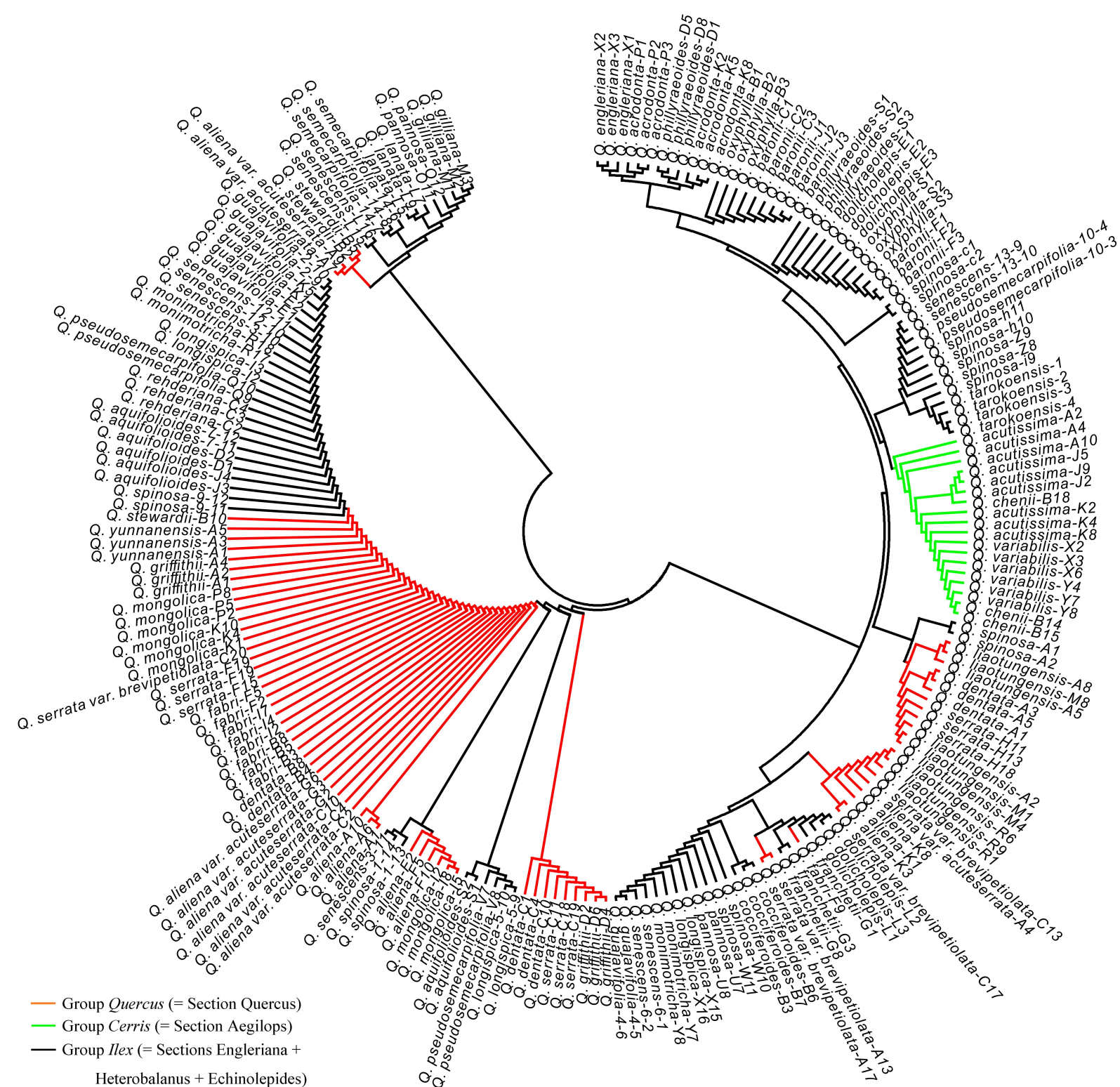

Supplement: Figure S8 — NJ tree of the single barcode candidate ycf1 region for the Chinese oak species in Quercus subgenus Quercus. [file Image8.PDF]

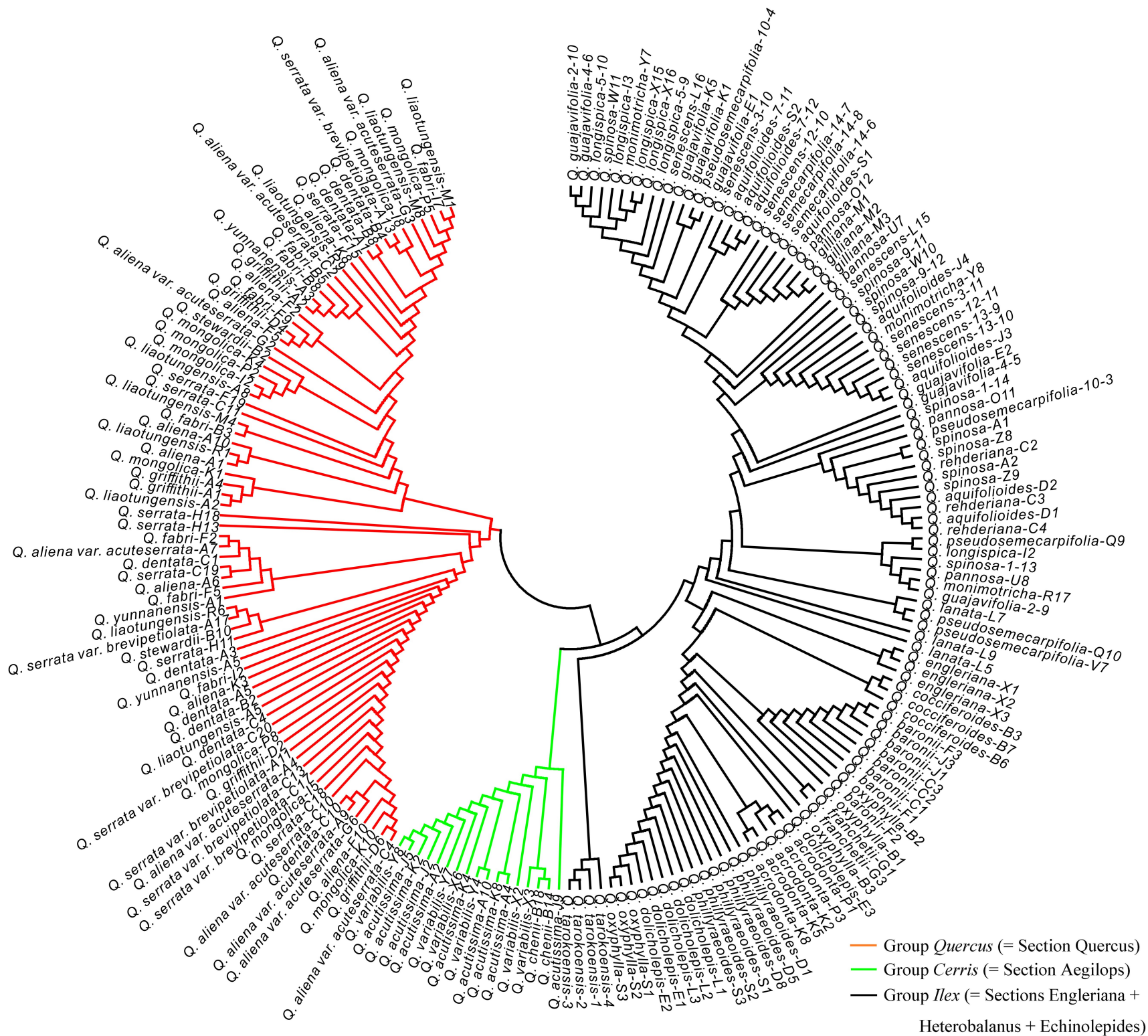

Supplement: Figure S9 — NJ tree of the single barcode ITS region for the Chinese oak species in Quercus subgenus Quercus. [file Image9.PDF]

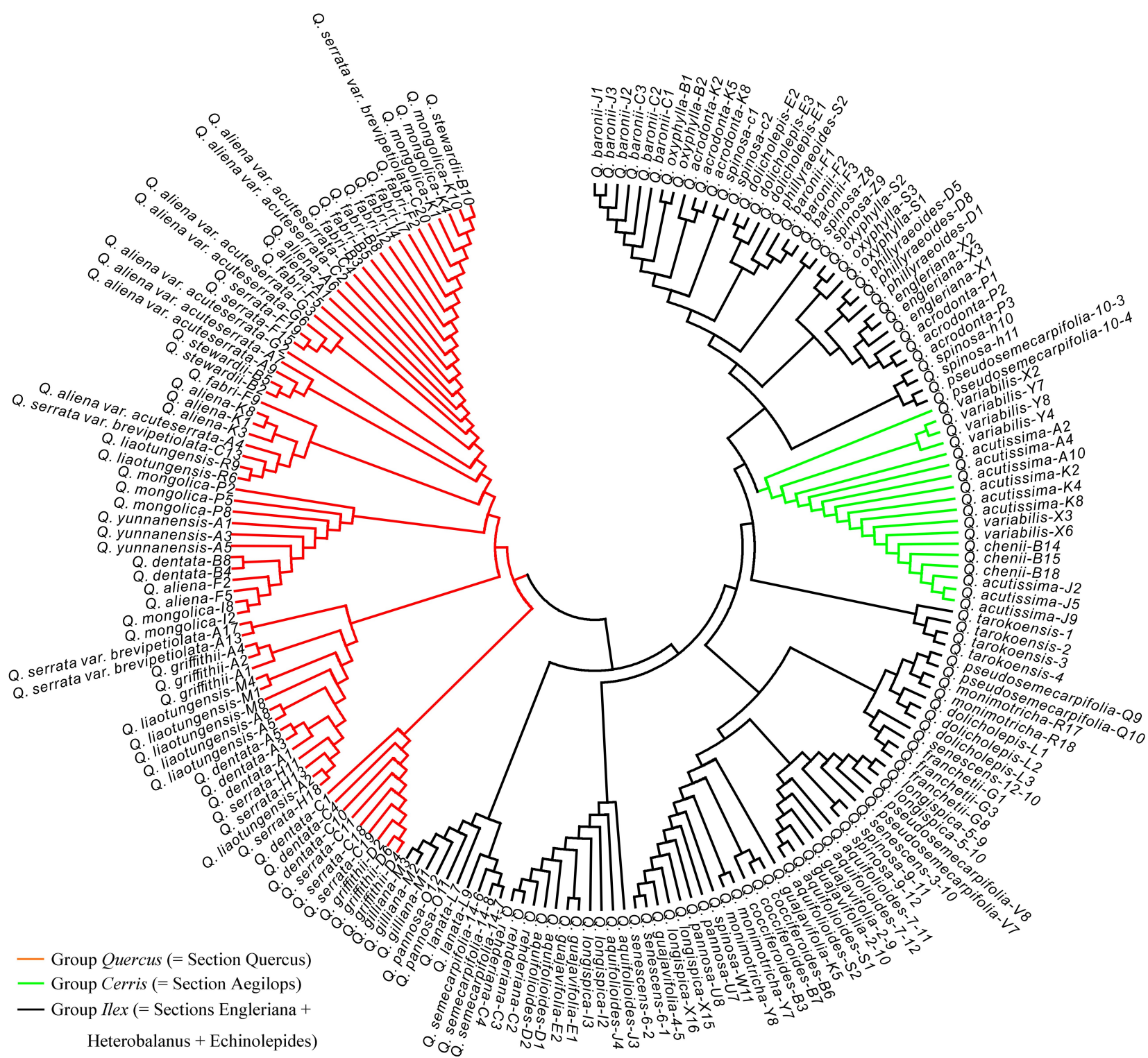

Supplement: Figure S11 — NJ tree based on the combination of five cpDNA barcode candidates for the Chinese oak species in Quercus subgenus Quercus. [file Image11.PDF]

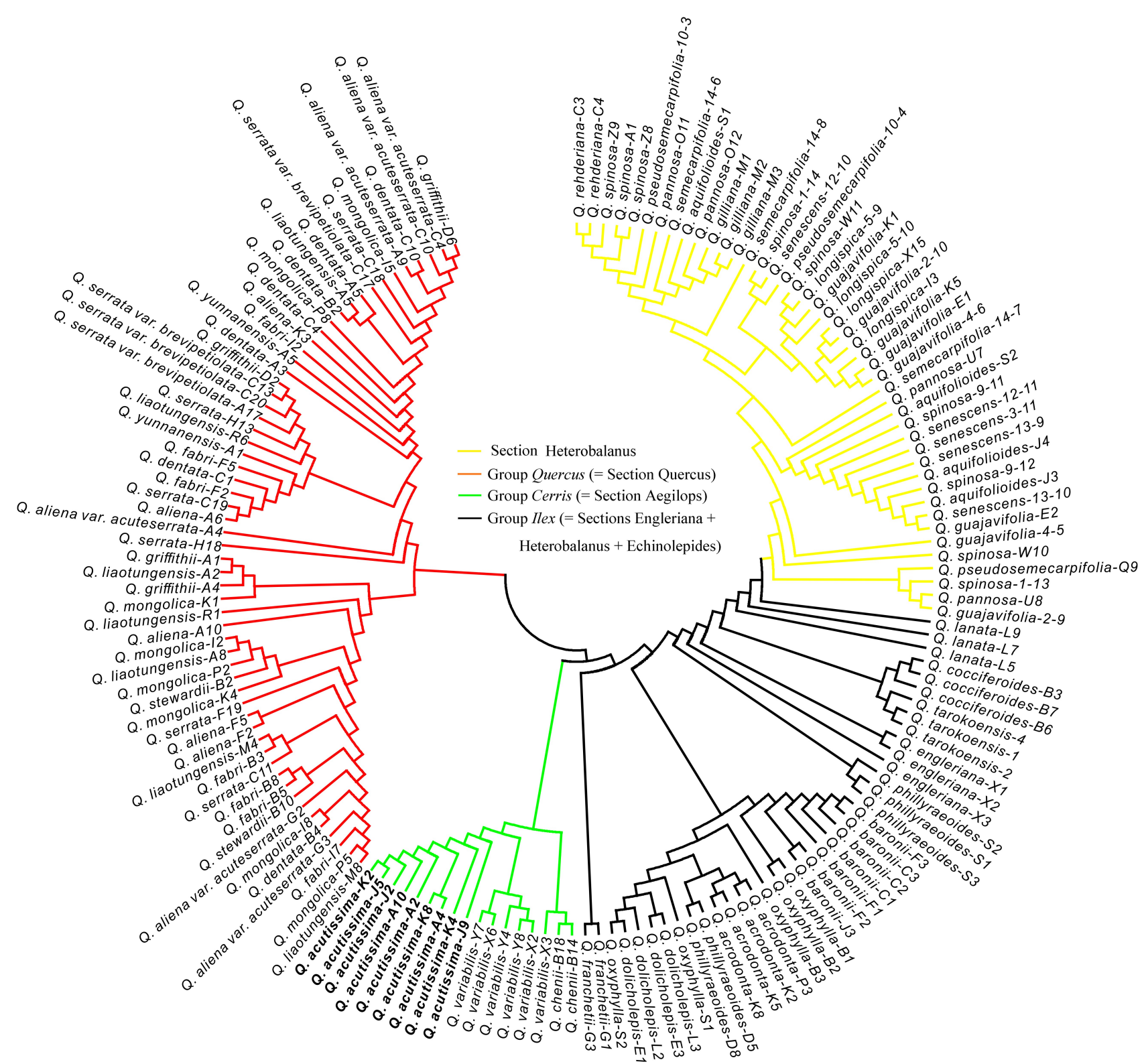

Supplement: Figure S12 — NJ tree based on the combination of two nuclear genes for the Chinese oak species in Quercus subgenus Quercus highlights the species discrimination of Quercus acutissima from Quercus variabilis + Quercus chenii in Group Cerris and shows the monophyletic subclade of Section Heterobalanus in Group Ilex. [file Image12.PDF]

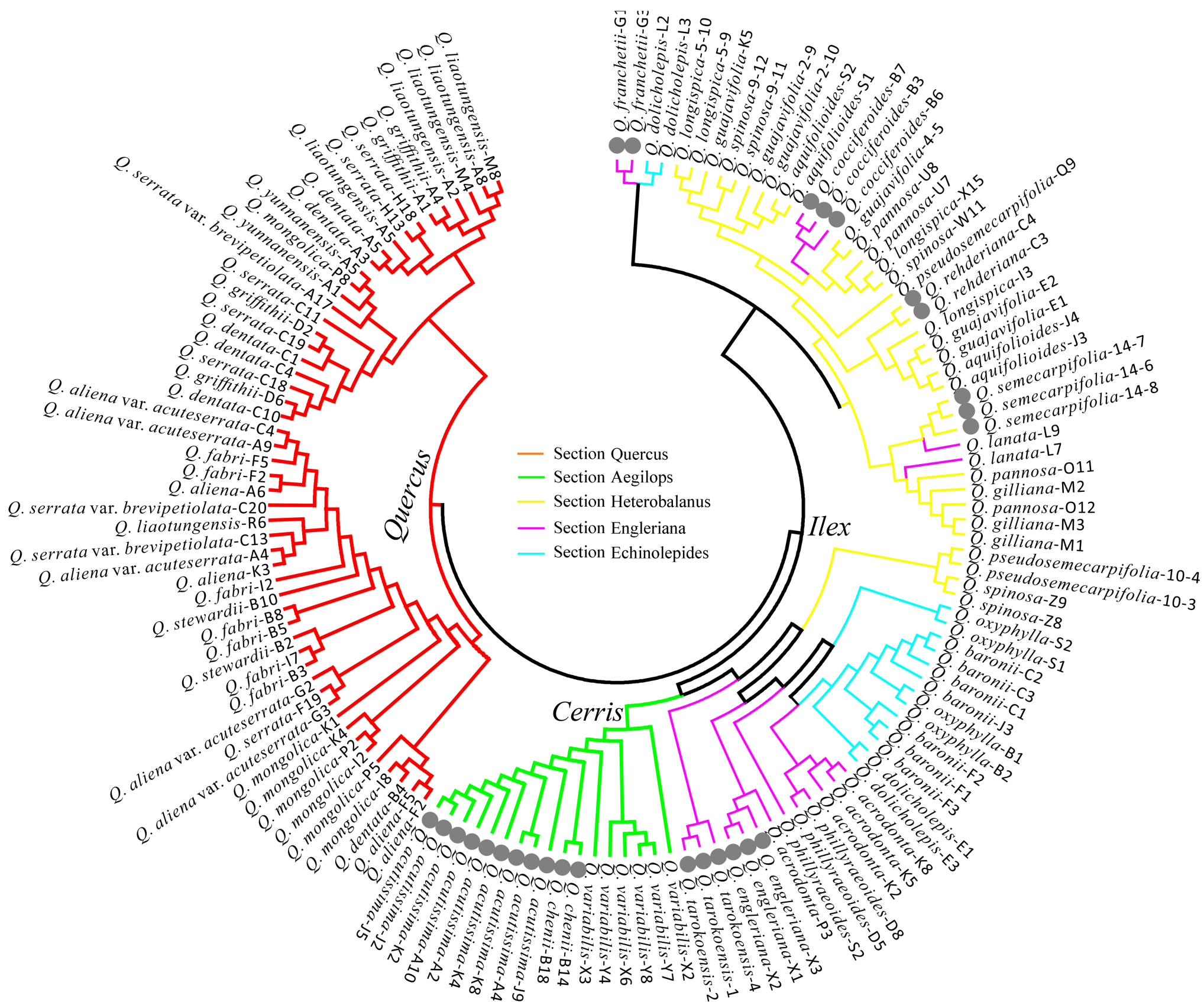

Supplement: Figure S13 — NJ tree of the combination with all seven candidate barcodes shows the phylogenetic implications for the Chinese oak species in Quercus subgenus Quercus. Gray dots highlight the individuals of identified oak species. [file Image13.PDF]
