# Supplementary material for: Development of Chloroplast and Nuclear DNA Markers for Chinese Oaks (Quercus Subgenus Quercus) and Assessment of Their Utility as DNA Barcodes
Source: Front Plant Sci. 2017 May 19;8:816. doi: 10.3389/fpls.2017.00816 (PMC5437370; doi:10.3389/fpls.2017.00816)
Supplement: Figure S6 — NJ tree of the single barcode candidate ycf3-trnS region for the Chinese oak species in Quercus subgenus Quercus. [file Image6.PDF]

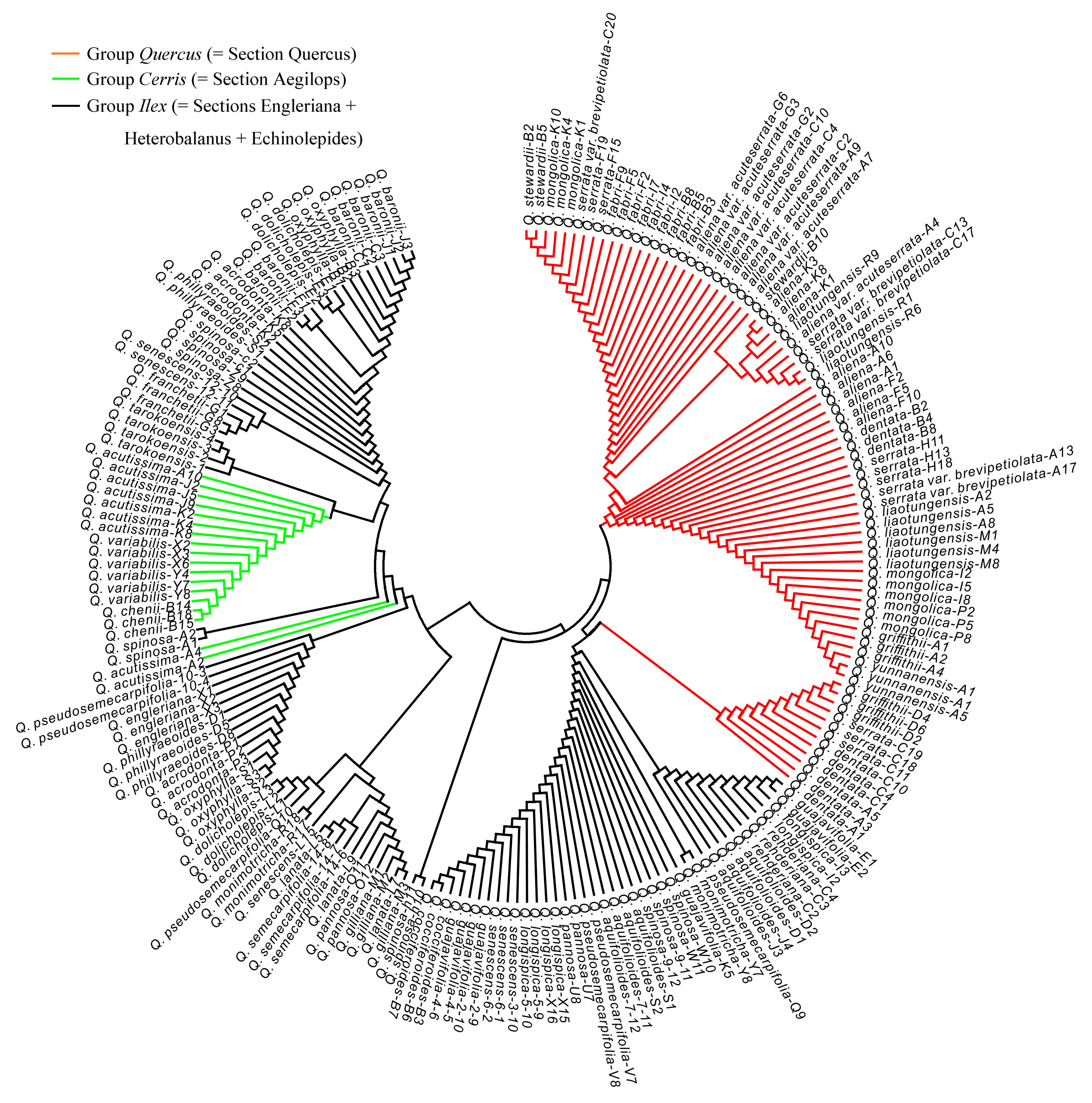
Group *Quercus* (= Section Quercus)
Group *Cerris* (= Section Aegilops)
Group *Ilex* (= Sections Engleriana + Heterobalanus + Echinolepides)
