# Supplementary material for: Development of Chloroplast and Nuclear DNA Markers for Chinese Oaks (Quercus Subgenus Quercus) and Assessment of Their Utility as DNA Barcodes
Source: Front Plant Sci. 2017 May 19;8:816. doi: 10.3389/fpls.2017.00816 (PMC5437370; doi:10.3389/fpls.2017.00816)
Supplement: Table S1 — Information of voucher specimens of the 35 Chinese oak species in Quercus subgenus Quercus. [file Table1.DOCX]

| **Table S1** Information of voucher specimens of the 35 Chinese oak species in *Quercus* subgenus *Quercus* | | | | | |  |  |  |  |  |  |
| --- | --- | --- | --- | --- | --- | --- | --- | --- | --- | --- | --- |
| Group | Section | Species | Voucher ID |  |  |  |  |  | Collector |  |  |
| *Quercus* | Quercus | *Quercus aliena* | SQHL20120526 | SQHL20130869 | SQHL20140705 |  |  |  | Dr. Jia Yang* | Dr. Li Feng* | Dr. Tao Zhou* |
|  |  | *Quercus aliena* var. *acutiserrata* | SQHL20110764 | SQHL20140780 | SQHL20140789 |  |  |  | Dr. Jia Yang | Dr. Xiao Zhang* | Dr. Li Feng |
|  |  | *Quercus dentata* | SQHL20110765 | SQHL20110768 | SQHL20110874 |  |  |  | Dr. Jia Yang | Dr. Tao Zhou |  |
|  |  | *Quercus fabri* | SQHL20100827 | SQHL20100833 | SQHL20100853 |  |  |  | Dr. Jia Yang | Dr. Zhaohui Huang* | |
|  |  | *Quercus serrata* | SQHL20100708 | SQHL20100709 | SQHL20100714 |  |  |  | Dr. Jia Yang | Dr. Tao Zhou |  |
|  |  | *Quercus serrata* var. *brevipetiolata* | SQHL20100837 | SQHL20100930 |  |  |  |  | Dr. Jia Yang | Xu Meng* |  |
|  |  | *Quercus liaotungensis* | SQHL20100731 | SQHL20100735 | SQHL20100762 |  |  |  | Dr. Jia Yang | Xiaodan Chen* |  |
|  |  | *Quercus mongolica* | SQHL20110783 | SQHL20110811 | SQHL20110815 |  |  |  | Dr. Jia Yang | Dr. Panfeng Dai^#^ |  |
|  |  | *Quercus griffithii* | SQHL20140898 | SQHL201009123 |  |  |  |  | Dr. Jia Yang |  |  |
|  |  | *Quercus yunnanensis* | SQHL20100834 |  |  |  |  |  | Dr. Jia Yang |  |  |
|  |  | *Quercus stewardii* | SQHL20100828 |  |  |  |  |  | Dr. Jia Yang |  |  |
| *Cerris* | Aegilops | *Quercus acutissima* | SAML20100902 | SAML20110709 | SAML20110838 |  |  |  | Dr. Zhaohui Huang | Dr. Jia Yang | Dr. Li Feng |
|  |  | *Quercus variabilis* | SAML20100704 | SAML20100841 |  |  |  |  | Dr. Jia Yang | Xu Meng |  |
|  |  | *Quercus chenii* | SAML20100901 |  |  |  |  |  | Dr. Jia Yang |  |  |
| *Ilex* | Heterobalanus | *Quercus spinosa* | SBCY20100910 | SBCY20140604 | SBCY20110637 | SBCY20110824 | SBCY20110908 | SBCY20121009 | Dr. Li Feng | Dr. Jia Yang |  |
|  |  | *Quercus aquifolioides* | SBCY20100911 | SBCY20100912 | SBCY20100915 | SBCY20100916 |  |  | Dr. Jia Yang | Dr. Li Feng |  |
|  |  | *Quercus rehderiana* | SBCY20100839 |  |  |  |  |  | Dr. Jia Yang |  |  |
|  |  | *Quercus pseudosemecarpifolia* | SBCY20100841 | SBCY20110954 |  |  |  |  | Dr. Jia Yang | Xu Meng |  |
|  |  | *Quercus pannosa* | SBCY20100913 | SBCY20100948 |  |  |  |  | Dr. Li Feng |  |  |
|  |  | *Quercus longispica* | SBCY20101017 | SBCY20101044 | SBCY20101050 |  |  |  | Dr. Jia Yang |  |  |
|  |  | *Quercus monimotricha* | SBCY20100914 | SBCY20100922 |  |  |  |  | Dr. Jia Yang | Xu Meng |  |
|  |  | *Quercus senescens* | SBCY20100934 | SBCY20100935 | SBCY20101047 |  |  |  | Dr. Li Feng |  |  |
|  |  | *Quercus guajavifolia* | SBCY20100119 | SBCY20100920 | SBCY20100918 |  |  |  | Dr. Jia Yang | Dr. Zhaohui Huang | |
|  |  | *Quercus semecarpifolia* | SBCY20101045 |  |  |  |  |  | Dr. Li Feng | Dr. Jia Yang |  |
|  |  | *Quercus gilliana* | SBCY20101036 |  |  |  |  |  | Dr. Jia Yang |  |  |
|  | Engleriana | *Quercus engleriana* | SEBD20130838 |  |  |  |  |  | Dr. Jia Yang | Dr. Tao Zhou |  |
|  |  | *Quercus cocciferoides* | SEBD20120812 |  |  |  |  |  | Dr. Jia Yang |  |  |
|  |  | *Quercus phillyraeoides* | SEBD20130840 | SEBD20140911 |  |  |  |  | Dr. Li Feng | Dr. Jia Yang |  |
|  |  | *Quercus franchetii* | SEBD20110934 |  |  |  |  |  | Dr. Jia Yang | Dr. Tao Zhou |  |
|  |  | *Quercus acrodonta* | SEBD20110833 | SEBD20120813 |  |  |  |  | Dr. Jia Yang | Dr. Zhaohui Huang | |
|  |  | *Quercus lanata* | SEBD20111028 |  |  |  |  |  | Dr. Jia Yang |  |  |
|  |  | *Quercus tarokoensis* | SEBD20110623 |  |  |  |  |  | Dr. Ching-I Peng^a^ | Dr. Zhikai Yang^b^ | Dr. Weixin Hu^c^ |
|  | Echinolepides | *Quercus dolicholepis* | SEJZ20110714 | SEJZ20150612 |  |  |  |  | Dr. Jia Yang |  |  |
|  |  | *Quercus oxyphylla* | SEJZ20110713 | SEJZ20120826 |  |  |  |  | Dr. Panfeng Dai | Dr. Li Feng |  |
|  |  | *Quercus baronii* | SEJZ20110706 | SEJZ20110708 | SEJZ20140725 |  |  |  | Dr. Jia Yang | Dr. Tao Zhou |  |
| *: College of Life Sciences, Northwest University | | |  |  |  |  |  |  |  |  |  |
| ^#^: College of Agriculture, Henan University of Science and Technology | | | |  |  |  |  |  |  |  |  |
| ^a^: Biodiversity Research Center, Academia Sinica (BRCAS) of Taiwan | | | |  |  |  |  |  |  |  |  |
| ^b^: National Taiwan University | | |  |  |  |  |  |  |  |  |  |
| ^c^: National Museum of Natural Science (NMNH) of Taiwan | | |  |  |  |  |  |  |  |  |  |
