# Supplementary material for: Development of Chloroplast and Nuclear DNA Markers for Chinese Oaks (Quercus Subgenus Quercus) and Assessment of Their Utility as DNA Barcodes
Source: Front Plant Sci. 2017 May 19;8:816. doi: 10.3389/fpls.2017.00816 (PMC5437370; doi:10.3389/fpls.2017.00816)
Supplement: Table S2 — Information of 49 designed cpDNA markers based on the comparison of two chloroplast genomes of Quercus rubra and Castanea mollissima. [file Table2.DOCX]

| **Table S2** Information of 49 designed cpDNA markers based on the comparison of two chloroplast genomes of *Quercus rubra* and *Castanea mollissima* | | | | | |  |
| --- | --- | --- | --- | --- | --- | --- |
| Primer ID | Amplification region | Forward primer (5'-3') | Reverse primer (5'-3') | Repeat unit | Production size (bp) | Amplification success (%) |
| B1 | *psb*A-*mat*K | GACGGTTTTCAGTGCTGGTTATCC | TTTTCATCAATGGTCTGTCC | - | 666 | 100 |
| B2 | *mat*K-*trn*K^UUU^ | TCTTACGATTTCTGCCCCTTCT | TTCTTAGCGGATCGGTTCAAAA | - | 942 | 100 |
| B3 | *rps*16 | TTGGGATAGATGTAGATGAATAA | TCGGGGGTTGGGATGTAAATAGT | - | 768 | 100 |
| B4 | *mat*K | ACCCAGTCCATCTGGAAATCTTGGTTC | CGTACAGTACTTTTGTGTTTACGAG | - | 853 | 100 |
| B5 | *trn*G^GCC^-*trn*R^UCU^ | CATGGATAAATAGAGAAGGGAGTA | ATATAAGCGGGTAGCGGGAATC | - | 511 | 100 |
| B6 | *atp*A-*atp*F | ACCGCCACCCATTAATACAACAC | AAAGGGGCTCTAGGAACTCTGACT | - | 410 | 100 |
| B7 | *atp*F^(5')^ | TGGCTCCCCTGCGTAGTTCTT | TAGCATTTCGCGATTCATTGGTAA | - | 747 | 0 |
| B8 | *atp*F-*atp*H | CCATAAACCCAAGGAAAGGAAAGA | TGGACTGGTTGTAGCGTTAGCACT | - | 508 | 0 |
| B9 | *atp*I-*rps*2 | ATAATGGCTGAACCTAATAAGATA | TCCCAGCAAATGATGACG | - | 480 | 86 |
| B10 | *rpo*C1^(5')^ | TATGGGCCTAGCAAAAGAAAAAT | AAGGACTGGTATACTGCGATGTGT | - | 879 | 0 |
| B11 | *rpo*B^(3')^ | CGGATACTCGGGGTCAAATAC | GCGGATGGTGCTGCTACA | - | 852 | 100 |
| B12 | *pet*N-*psb*M | CTTTACATTTTCTCTTTCACTCGT | GACTGTTTCGTTCTTATCAATG | - | 1021 | 0 |
| B13 | *trn*D^GUC^-*trn*E^UUC^ | CTTGACAGGGCGGTGCTCT | TAATGGGGACGGACTGTAAAT | - | 577 | 100 |
| B14 | *psb*C-*lhb*A | ATTGATCGCGATTTTGAACCT | CCACGAATCTATTAATGCTGTATG | - | 675 | 100 |
| B15 | *lhb*A-*trn*fM^CAU^ | AAAAATGAAATGATCCCCTCCTAA | ATCCCGTCTCCGCAACATTTT | - | 914 | 0 |
| B16 | *psa*A-*ycf*3 | TCCGGCGAACGAATAATCAT | CAAGGCGTTTCGAATAGGAGCACT | - | 781 | 0 |
| B17 | *ycf*3-*trn*S^GGA^ | AAATCGCACCATCTCTGTAATAGG | CAAAACCGGGTGAATAGTGAGTC | - | 1068 | 86 |
| B18 | *trn*L^UAA^-*trn*F^GAA^ | TTAAAAATGGGCAATCCTGAGC | TTATCCGTGCATCGTCCTTATTTT | - | 846 | 93 |
| B19 | *ndh*J-*ndh*K | CCAATCCCCAGGCTTTAT | GATCGAATTAGGTCTCAACAGG | - | 447 | 0 |
| B20 | *ndh*C-*trn*V^UAC^ | CCATTGGTTCTATTCCCGATTCAT | CTAGCCGATATTTCTTTCAGTTTA | - | 753 | 0 |
| B21 | *trn*V^UAC^-*atp*E | TTCCCGAAAACATAGAGTAAGACA | AGCTGAAGGCAAGAGACAAACAAT | - | 669 | 100 |
| B22 | *rbc*L | CAAGTATGGCCGCCCCCTATT | AGCAAGATCACGTCCCTCATT | - | 826 | 100 |
| B23 | *acc*D-*psa*L | GCTCCACGCTTTCTTTTCTC | CCTGGATTTACTATTTTTACCTGT | - | 790 | 0 |
| B24 | *ycf*4-*cem*A | GGCGCTATTCCTTTGACTCGTA | ATGCAAGATATAGAAGGGGAATGA | - | 778 | 0 |
| B25 | *pet*A-*psb*J | GAGCCTTTTTCTTTTCTACTA | AACCCCTCTTTATTTATCTG | - | 882 | 0 |
| B26 | *pet*L-*trn*W^CCA^ | CAGCTCTATTTATCGGGTTGAA | TCTTAGGTCGAAAATTACACTGAA | - | 466 | 100 |
| B27 | *psa*J-*rps*18 | CTCCCCTTCCCATCTTTTCATTTT | GTCGGGGATTTCTTTTTGGTTTGT | - | 764 | 0 |
| B28 | *rps*18-*rpl*20 | ATTAGTCGATTTATTAGTGAACA | ATCGGGATAGAGGTAGGCAAAAG | - | 707 | 100 |
| B29 | *rps*11-*rps*8 | TATTCTACGCGCACTCTTACG | AACGGGTTTCTATTCTCACTCTC | - | 740 | 100 |
| B30 | *rps*3-*rps*19 | TGCAAACCAAAGAGAATGATGAC | TCCGAGGACACGCAAAAA | - | 692 | 100 |
| B31 | *ndh*F^(3')^ | TTCGGCCAATGCTCTTAT | TCCACCCCTTGCCTGTTTT | - | 847 | 100 |
| B32 | *ndh*F-*rpl*32 | TGTTTCGGATTCGCCAGTT | TCCAATCAAGAAGTTAGAAT | - | 770 | 0 |
| B33 | *ccs*A-*ndh*D | AAATACAAACAAGCATGGGACAGC | TCAACGGAGCCTTTTTACAA | - | 838 | 0 |
| B34 | *ndh*D-*ndh*E | AAATAATTCGTAGTAAAAACAAGA | AGCATTAATCGATCCAGAAGCAGA | - | 525 | 0 |
| B35 | *ndh*A | CTTCACTGTTTATCGTCGCTATTA | TCTTTTTATTCATTATTCGGATTA | - | 938 | 0 |
| B36 | *rps*15-*ycf*1 | ATCCCCTGTTTTCTTCTTTTTC | CAATAATGGTAGTCGTTTCAGTCT | - | 575 | 100 |
| B37 | *ycf*1^(5')^ | CGCGATAGGGTCCGTTCA | CTATTTAGGCAGAGTACCGTCACC | - | 839 | 93 |
| B38 | *ycf*1 | ATTCTGATGGTCCGGAAGGG | CCTTATCAGACTGAAACGACTAC | - | 546 | 100 |
| B39 | *trn*H^GUG^-*psb*A | GTTATGCATGAACGTAATGCTC | CGCGCATGGTGGATTCACAATCC | - | 665 | 100 |
| R1 | *rps*12-*trn*V^GAC^ | CCAAATTGACGGGTTAGTGTGA | TTACCTCCGCGGAAAAGATGAT | ttttctattatattagta | 501 | 100 |
| R2 | *clp*P | TTGTTTTGGCCTGTTTACTTTAC | AGATGCATCGTGGGTTGAC | atattcaatgtattcatt | 688 | 100 |
| R3 | *rpl*2 | GCGGATTTCCCCTCTTTTG | GACGGGTTATTCACATTTCATTTG | tcttatatatatcgtataat | 560 | 100 |
| R5 | *ycf*2 | ATATACGATCAACCAACATTTA | GATTTCCTGCGATTATTTCTA | tttttgtccaagttacttctt | 639 | 100 |
| R7 | *rps*16-*trn*Q^UUG^ | AAAGAAAATGATGGATGTAAGAAT | TAATGTAATGATAGTGAAATAGGA | atatatatataataaataatattta | 531 | 100 |
| R8 | *lhb*A-*trn*G^UCC^ | TGCTTCTCCTGATGGTTGG | TCAGATACTCCTTTGTTTTTCAGA | ataattttattatactatttattt | 709 | 0 |
| R9 | *rpl*32-*trn*L^UAG^ | AACGCTCAATCGCTTTTCTAA | GAGCAGCGTGTCTACCGATTTC | tatttttcaacattttatttcttact | 709 | 50 |
| R10 | *ycf*3-*trn*S^GGA^ | TTGTCTAAGAATGAAAGGAACTA | CATTACAATATTTATACCAGAAGA | atacaatcaaatagaaaa | 481 | 100 |
