# Supplementary material for: Development of Chloroplast and Nuclear DNA Markers for Chinese Oaks (Quercus Subgenus Quercus) and Assessment of Their Utility as DNA Barcodes
Source: Front Plant Sci. 2017 May 19;8:816. doi: 10.3389/fpls.2017.00816 (PMC5437370; doi:10.3389/fpls.2017.00816)
Supplement: Table S3 — Estimation of intraspecific genetic diversity (π) for 14 deciduous oak species of morphology-based Sections Quercus and Aegilops based on the designed cpDNA markers. [file Table3.DOCX]

| **Table S3** Estimation of intraspecific genetic diversity (π) for 14 deciduous oak species of morphology-based Sections Quercus and Aegilops based on the designed cpDNA markers | | | | | | | | | | | | | | | |  |  |
| --- | --- | --- | --- | --- | --- | --- | --- | --- | --- | --- | --- | --- | --- | --- | --- | --- | --- |
|  | Quercus |  |  |  |  |  |  |  |  |  |  |  | Aegilops |  |  |  |  |
| Marker ID | *Q. aliena* | *Q. aliena* var. *acuteserrata* | *Q. dentata* | *Q. fabri* | *Q. serrata* | *Q. serrata* var. *brevipetiolata* | *Q. liaotungensis* | *Q. mongolica* | *Q. griffithii* | *Q. yunnanensis* | *Q. stewardii* | | *Q. acutissima* | *Q. variabilis* | *Q. chenii* |  | Mean diversity (π) |
| B1 | 0.0000 | 0.0000 | 0.0013 | 0.0000 | 0.0026 | 0.0039 | 0.0013 | 0.0013 | 0.0049 | 0.0013 | 0.0026 |  | 0.0000 | 0.0020 | Null |  | 0.0016 |
| B2 | 0.0035 | 0.0013 | 0.0027 | 0.0000 | 0.0013 | 0.0000 | 0.0013 | Null | 0.0000 | Null | 0.0000 |  | 0.0000 | 0.0000 | 0.0000 |  | 0.0008 |
| B3 | 0.0011 | 0.0011 | 0.0011 | 0.0000 | 0.0011 | 0.0000 | 0.0016 | 0.0000 | 0.0033 | 0.0000 | 0.0022 |  | 0.0000 | 0.0019 | 0.0040 |  | 0.0012 |
| B4 | 0.0000 | 0.0000 | 0.0000 | 0.0000 | 0.0000 | 0.0000 | 0.0000 | 0.0000 | 0.0000 | 0.0000 | 0.0010 |  | 0.0000 | 0.0029 | 0.0000 |  | 0.0003 |
| B5 | 0.0000 | 0.0000 | 0.0000 | 0.0000 | 0.0000 | 0.0017 | 0.0000 | 0.0000 | 0.0000 | 0.0000 | 0.0000 |  | 0.0017 | 0.0000 | 0.0000 |  | 0.0002 |
| B6 | 0.0000 | 0.0025 | 0.0000 | 0.0000 | 0.0000 | 0.0000 | 0.0000 | 0.0000 | 0.0025 | 0.0000 | 0.0000 |  | 0.0000 | 0.0000 | 0.0000 |  | 0.0004 |
| B9 | x | x | 0.0000 | 0.0000 | 0.0019 | 0.0019 | 0.0019 | 0.0019 | 0.0000 | 0.0000 | 0.0000 |  | 0.0000 | 0.0000 | 0.0000 |  | 0.0006 |
| B11 | 0.0000 | 0.0000 | 0.0000 | 0.0000 | 0.0000 | 0.0009 | 0.0000 | 0.0000 | 0.0000 | 0.0000 | 0.0000 |  | 0.0000 | 0.0000 | 0.0000 |  | 0.0001 |
| B13 | 0.0042 | 0.0069 | 0.0083 | 0.0014 | 0.0111 | 0.0035 | 0.0042 | 0.0055 | 0.0055 | 0.0056 | 0.0028 |  | 0.0097 | 0.0093 | 0.0000 |  | 0.0056 |
| B14 | 0.0031 | 0.0015 | 0.0015 | 0.0015 | 0.0046 | 0.0015 | 0.0000 | 0.0000 | 0.0015 | 0.0015 | 0.0000 |  | 0.0031 | 0.0058 | 0.0000 |  | 0.0018 |
| B17 | 0.0030 | 0.0000 | 0.0015 | 0.0008 | 0.0015 | 0.0023 | 0.0023 | 0.0023 | 0.0021 | 0.0000 | 0.0000 |  | 0.0000 | 0.0000 | 0.0000 |  | 0.0011 |
| B18 | x | Null | 0.0000 | Null | 0.0000 | 0.0000 | 0.0010 | 0.0010 | 0.0000 | Null | 0.0000 |  | 0.0000 | 0.0059 | Null |  | 0.0009 |
| B21 | 0.0000 | Null | 0.0000 | 0.0000 | 0.0000 | 0.0011 | 0.0000 | 0.0000 | 0.0000 | 0.0000 | 0.0000 |  | 0.0184 | 0.0097 | Null |  | 0.0024 |
| B22 | 0.0000 | 0.0000 | 0.0010 | 0.0000 | 0.0000 | 0.0019 | 0.0010 | 0.0000 | 0.0000 | 0.0000 | 0.0000 |  | 0.0038 | 0.0000 | 0.0000 |  | 0.0005 |
| B26 | 0.0017 | 0.0000 | Null | Null | 0.0000 | 0.0000 | 0.0017 | 0.0017 | 0.0000 | 0.0000 | 0.0000 |  | 0.0017 | 0.0000 | 0.0017 |  | 0.0006 |
| B28 | 0.0011 | 0.0011 | 0.0011 | 0.0000 | 0.0000 | 0.0011 | 0.0021 | 0.0000 | 0.0011 | 0.0000 | 0.0000 |  | 0.0000 | 0.0000 | 0.0000 |  | 0.0005 |
| B29 | 0.0030 | 0.0010 | 0.0030 | 0.0030 | 0.0046 | 0.0000 | 0.0040 | 0.0030 | 0.0038 | 0.0000 | 0.0020 |  | 0.0010 | 0.0010 | 0.0010 |  | 0.0022 |
| B30 | 0.0022 | 0.0011 | 0.0033 | 0.0000 | 0.0044 | 0.0000 | 0.0011 | 0.0022 | 0.0022 | 0.0000 | 0.0011 |  | 0.0000 | 0.0000 | 0.0000 |  | 0.0013 |
| B31 | 0.0000 | 0.0017 | 0.0000 | 0.0000 | 0.0013 | 0.0009 | 0.0017 | 0.0000 | 0.0009 | 0.0000 | 0.0009 |  | 0.0009 | 0.0017 | 0.0009 |  | 0.0008 |
| B36 | 0.0041 | 0.0081 | 0.0068 | 0.0081 | 0.0061 | 0.0027 | 0.0041 | 0.0027 | 0.0027 | 0.0014 | 0.0081 |  | 0.0061 | 0.0101 | 0.0000 |  | 0.0051 |
| B37 | 0.0009 | 0.0000 | 0.0018 | 0.0000 | 0.0027 | 0.0000 | 0.0018 | 0.0009 | 0.0018 | 0.0000 | 0.0000 |  | Null | x | 0.0000 |  | 0.0008 |
| B38 | 0.0000 | 0.0015 | 0.0000 | 0.0000 | 0.0000 | 0.0000 | 0.0000 | 0.0000 | 0.0000 | 0.0000 | 0.0046 |  | 0.0030 | 0.0000 | 0.0000 |  | 0.0007 |
| B39 | 0.0000 | 0.0000 | 0.0000 | 0.0000 | 0.0000 | 0.0000 | 0.0000 | 0.0000 | 0.0000 | 0.0000 | 0.0021 |  | 0.0000 | 0.0021 | 0.0000 |  | 0.0042 |
| x: PCR amplification failure; Null: Intraspecific diversity of species with amplicons less than two are not estimated | | | | | | | | | |  |  |  |  |  |  |  |  |
