# Supplementary material for: Development of Chloroplast and Nuclear DNA Markers for Chinese Oaks (Quercus Subgenus Quercus) and Assessment of Their Utility as DNA Barcodes
Source: Front Plant Sci. 2017 May 19;8:816. doi: 10.3389/fpls.2017.00816 (PMC5437370; doi:10.3389/fpls.2017.00816)
Supplement: Table S4 — Estimation of genetic information and Automatic Barcode Gap Discovery (ABGD) results for nine candidate barcodes based on 14 Chinese oak species. [file Table4.DOCX]

| **Table S4** Estimation of genetic information and Automatic Barcode Gap Discovery (ABGD) results for nine candidate barcodes based on 14 Chinese oak species | | | | | | | |
| --- | --- | --- | --- | --- | --- | --- | --- |
| Primer ID | Amplification region | No. variable sites | No. indels | No. diagnostic characters | No. species groups | Intraspecific distance range (SE) | Interspecific distance range (SE) |
| B2 | *mat*K-*trn*K^UUU^ | 30 | 0 | 1 | 1 | 0~0.0040 (0.0022) | 0~0.0358 (0.0064) |
| B3 | *rps*16 | 20 | 3 | 1 | 1 | 0~0.0066 (0.0032) | 0~0.0146 (0.0045) |
| B4 | *mat*K | 6 | 0 | 1 | 1 | 0~0.0057 (0.0029) | 0~0.0057 (0.0028) |
| B17 | *ycf*3-*trn*S^GGA^ | 10 | 4 | 1 | 1 | 0~0.0049 (0.0027) | 0.0004 (0.0004)~0.0046 (0.0022) |
| B31 | *ndh*F | 20 | 1 | 1 | 1 | 0~0.0026 (0.0019) | 0~0.0202 (0.0047) |
| B36 | *rps*15-*ycf*1 | 27 | 0 | 6 | 2 | 0~0.0162 (0.0054) | 0.0032 (0.0019)~0.0358 (0.0082) |
| B37 | *ycf*1^(5')^ | 23 | 5 | 2 | 1 | 0~0.0042 (0.0023) | 0~0.0254 (0.0058) |
| B38 | *ycf*1 | 12 | 0 | 1 | 3 | 0~0.0068 (0.0039) | 0~0.0114 (0.0049) |
| B39 | *trn*H^GUG^-*psb*A | 5 | 3 | 3 | 2 | 0~0.0032 (0.0031) | 0~0.0144 (0.0063) |
