# Supplementary material for: Development of Chloroplast and Nuclear DNA Markers for Chinese Oaks (Quercus Subgenus Quercus) and Assessment of Their Utility as DNA Barcodes
Source: Front Plant Sci. 2017 May 19;8:816. doi: 10.3389/fpls.2017.00816 (PMC5437370; doi:10.3389/fpls.2017.00816)
Supplement: Table S5 — Differentiation tests between the minimum interspecific and maximum intraspecific genetic distance (p-distance) for each barcode candidate and section. [file Table5.DOCX]

| **Table S5** Differentiation tests between the minimum interspecific and maximum intraspecific genetic distance (p-distance) for each barcode candidate and section | | | | | | | | |
| --- | --- | --- | --- | --- | --- | --- | --- | --- |
|  |  | Student's t test | |  | Wilcoxon rank sum test | |  | Result |
|  |  | t statistic | P-value |  | W statistic | P-value |  |  |
| Barcode | *mat*K-*trn*K | 0.369 | = 0.712 |  | 2282.5 | = 0.626 |  | no difference |
|  | *ycf*3-*trn*S | 2.31 | < 0.05 |  | 1854.5 | < 0.05 |  | intra > inter |
|  | *psb*A-*trn*H | 0.907 | = 0.365 |  | 2123 | = 0.260 |  | no difference |
|  | *mat*K | 2.747 | < 0.05 |  | 1845 | < 0.05 |  | intra > inter |
|  | *ycf*1 | 3.003 | < 0.05 |  | 1514.5 | < 0.001 |  | intra > inter |
|  | ITS | 9.406 | < 0.001 |  | 707 | < 0.001 |  | intra > inter |
|  | SAP | 8.92 | < 0.001 |  | 677 | < 0.001 |  | intra > inter |
| Section | Quercus | 13.476 | < 0.001 |  | 5832 | < 0.001 |  | intra > inter |
|  | Aegilops | 2.46 | < 0.05 |  | 144.5 | < 0.05 |  | intra > inter |
|  | Heterobalanus | 8.57 | < 0.001 |  | 7826 | < 0.001 |  | intra > inter |
|  | Engleriana | -5.477 | < 0.001 |  | 1726 | < 0.001 |  | intra < inter |
|  | Echinolepides | 3.191 | < 0.05 |  | 103.5 | < 0.05 |  | intra > inter |
