# Supplementary material for: Development of Chloroplast and Nuclear DNA Markers for Chinese Oaks (Quercus Subgenus Quercus) and Assessment of Their Utility as DNA Barcodes
Source: Front Plant Sci. 2017 May 19;8:816. doi: 10.3389/fpls.2017.00816 (PMC5437370; doi:10.3389/fpls.2017.00816)
Supplement: Table S6 — Estimation of the highest species discrimination rates for four barcoding methods based on single and all possible barcode combinations. [file Table6.DOCX]

| **Table S6** Estimation of the highest species discrimination rates for four barcoding methods based on single and all possible barcode combinations | | | | | | | |
| --- | --- | --- | --- | --- | --- | --- | --- |
| Methods | Single-barcode | 2-barocdes | 3-barcodes | 4-barcodes | 5-barcodes | 6-barcodes | 7-barcodes |
| Genetic distance-based | 11.43% | 14.29% | 17.14% | 14.29% | 14.29% | 8.57% | 0.00% |
| BLAST-based | 30.30% | 36.36% | 45.45% | 39.39% | 39.39% | 36.36% | 36.36% |
| Character-based | 30.30% | 57.58% | 69.70% | 78.79% | 81.82% | 84.85% | 84.85% |
| Tree-based | 17.14% | 22.86% | 26.47% | 29.41% | 29.41% | 30.30% | 24.24% |
