# Supplementary material for: Development of Chloroplast and Nuclear DNA Markers for Chinese Oaks (Quercus Subgenus Quercus) and Assessment of Their Utility as DNA Barcodes
Source: Front Plant Sci. 2017 May 19;8:816. doi: 10.3389/fpls.2017.00816 (PMC5437370; doi:10.3389/fpls.2017.00816)
Supplement: Table S7 — Species resolution of seven single barcodes and all their possible combinations for the Chinese oaks based on four barcoding methods. [file Table7.DOCX]

| **Table S7** Species resolution of seven single barcodes and all their possible combinations for the Chinese oaks based on four barcoding methods | | | | | |
| --- | --- | --- | --- | --- | --- |
|  | Marker | Genetic distance-based | BLAST-based | Character-based | Tree-based |
| Single-barcode | A | 0.0286 | **0.3030** | 0.1212 | 0.0286 |
|  | M | **0.1143** | 0.2424 | 0.1818 | 0.0286 |
|  | S | 0.0286 | 0.2121 | 0.0909 | 0.0286 |
|  | K | 0.0286 | 0.2121 | 0.0909 | 0.0571 |
|  | Y | 0.0857 | 0.2424 | 0.1515 | 0.1429 |
|  | I | 0.0000 | 0.1818 | **0.3030** | **0.1714** |
|  | P | 0.0286 | 0.1818 | 0.2121 | 0.1143 |
| 2-barcodes | A+M | 0.1143 | 0.3333 | 0.2121 | 0.0571 |
|  | A+S | 0.0286 | 0.2121 | 0.1212 | 0.0857 |
|  | A+I | 0.0571 | 0.2121 | 0.4545 | **0.2286** |
|  | A+K | 0.0286 | 0.3030 | 0.1212 | 0.0571 |
|  | A+P | 0.0857 | 0.3333 | 0.3636 | 0.1714 |
|  | A+Y | 0.1143 | 0.2121 | 0.2727 | 0.1429 |
|  | M+S | 0.1143 | 0.2424 | 0.2121 | 0.0857 |
|  | M+I | 0.0857 | 0.2727 | 0.3333 | **0.2286** |
|  | M+K | 0.1143 | 0.2121 | 0.2121 | 0.0571 |
|  | M+P | 0.1143 | **0.3636** | 0.3939 | 0.2000 |
|  | M+Y | **0.1429** | 0.3030 | 0.3636 | 0.1429 |
|  | S+I | 0.0000 | 0.2424 | 0.3333 | 0.1429 |
|  | S+K | 0.0286 | 0.2121 | 0.0909 | 0.0571 |
|  | S+P | 0.0857 | 0.2121 | 0.2424 | 0.1429 |
|  | S+Y | 0.1143 | 0.2727 | 0.2727 | 0.1429 |
|  | I+P | 0.0000 | 0.3030 | **0.5758** | 0.1765 |
|  | K+I | 0.0286 | 0.2424 | 0.3030 | 0.1429 |
|  | K+P | 0.1143 | 0.2121 | 0.2424 | 0.1714 |
|  | K+Y | **0.1429** | 0.2121 | 0.2424 | 0.1714 |
|  | Y+I | 0.0571 | 0.2424 | 0.4242 | 0.2000 |
|  | Y+P | 0.0571 | 0.3030 | 0.4242 | 0.2000 |
| 3-barcodes | A+M+S | 0.1143 | 0.3636 | 0.2121 | 0.0857 |
|  | A+M+I | 0.0857 | 0.3333 | 0.3939 | 0.2571 |
|  | A+M+K | 0.1143 | 0.3333 | 0.1515 | 0.0571 |
|  | A+M+P | 0.0857 | 0.2727 | 0.2727 | 0.1714 |
|  | A+M+Y | 0.1429 | 0.2424 | 0.3030 | 0.1714 |
|  | A+S+I | 0.0286 | 0.3636 | 0.4545 | 0.2000 |
|  | A+S+K | 0.0286 | 0.3333 | 0.1212 | 0.0857 |
|  | A+S+P | 0.1143 | 0.3333 | 0.3939 | 0.2353 |
|  | A+S+Y | 0.1143 | 0.3333 | 0.3333 | 0.1429 |
|  | A+I+P | 0.0000 | 0.3636 | 0.6364 | 0.2286 |
|  | A+K+I | 0.0286 | 0.3333 | 0.4545 | 0.2286 |
|  | A+K+P | 0.0857 | 0.3333 | 0.2727 | 0.1714 |
|  | A+K+Y | 0.1143 | 0.2121 | 0.2424 | 0.1429 |
|  | A+Y+I | 0.0857 | 0.2121 | 0.3030 | 0.1714 |
|  | A+Y+P | 0.0571 | 0.2121 | 0.4545 | 0.2000 |
|  | M+S+I | **0.1714** | 0.2121 | 0.3636 | 0.2286 |
|  | M+S+K | 0.0000 | 0.2727 | 0.1212 | 0.0857 |
|  | M+S+P | 0.1429 | 0.3030 | 0.4545 | 0.2571 |
|  | M+S+Y | 0.1429 | 0.3333 | 0.3636 | 0.1429 |
|  | M+I+P | 0.0286 | 0.3333 | 0.6667 | 0.2353 |
|  | M+K+I | 0.1429 | 0.2121 | 0.3333 | 0.2286 |
|  | M+K+P | 0.1429 | 0.2121 | 0.2424 | 0.2000 |
|  | M+K+Y | 0.1429 | 0.2727 | 0.3030 | 0.1714 |
|  | M+Y+I | 0.0857 | 0.2121 | 0.3939 | 0.2286 |
|  | M+Y+P | 0.0857 | 0.2727 | 0.4545 | 0.2000 |
|  | S+I+P | 0.0571 | 0.2727 | 0.6667 | **0.2647** |
|  | S+K+I | 0.0286 | 0.2727 | 0.3939 | 0.1714 |
|  | S+K+P | 0.0857 | 0.2121 | 0.3636 | 0.2571 |
|  | S+K+Y | 0.1143 | 0.3030 | 0.2424 | 0.1429 |
|  | S+Y+I | 0.0571 | 0.3030 | 0.4545 | 0.1714 |
|  | S+Y+P | 0.1429 | 0.3030 | 0.3939 | 0.2000 |
|  | K+I+P | 0.0286 | 0.3030 | 0.6061 | 0.2353 |
|  | K+Y+I | 0.0571 | 0.3030 | 0.3030 | 0.2000 |
|  | K+Y+P | 0.0571 | **0.4545** | 0.3939 | 0.2286 |
|  | Y+I+P | 0.0286 | 0.2727 | **0.6970** | **0.2647** |
| 4-barcodes | A+M+S+I | 0.0571 | 0.3333 | 0.5152 | 0.2286 |
|  | A+M+S+K | 0.0286 | 0.3636 | 0.1515 | 0.0857 |
|  | A+M+S+P | 0.1429 | 0.3333 | 0.4242 | 0.1765 |
|  | A+M+S+Y | 0.0286 | 0.3636 | 0.3636 | 0.1714 |
|  | A+M+K+I | **0.1429** | **0.3939** | 0.4545 | 0.2286 |
|  | A+M+K+P | 0.1143 | 0.3636 | 0.2727 | 0.1714 |
|  | A+M+K+Y | 0.0571 | 0.3636 | 0.2727 | 0.1714 |
|  | A+M+P+I | 0.0571 | 0.3333 | 0.6667 | 0.2353 |
|  | A+M+Y+I | 0.0000 | 0.3333 | 0.3939 | 0.2286 |
|  | A+M+Y+P | 0.0857 | 0.3333 | 0.4545 | 0.2000 |
|  | A+S+K+I | 0.0286 | 0.3333 | 0.5455 | 0.2000 |
|  | A+S+K+P | 0.0857 | 0.2727 | 0.3333 | 0.2059 |
|  | A+S+K+Y | 0.0571 | 0.2727 | 0.3333 | 0.1429 |
|  | A+S+P+I | 0.0857 | 0.3030 | 0.7273 | 0.2727 |
|  | A+S+Y+I | 0.0286 | 0.2121 | 0.5152 | 0.1714 |
|  | A+S+Y+P | 0.1143 | 0.3030 | 0.4545 | 0.2353 |
|  | A+K+P+I | 0.0857 | 0.3030 | 0.6970 | 0.2647 |
|  | A+K+Y+I | 0.0286 | 0.3333 | 0.4242 | 0.1714 |
|  | A+K+Y+P | 0.0571 | 0.3333 | 0.4848 | 0.2286 |
|  | A+Y+P+I | 0.0857 | 0.3333 | 0.7273 | 0.2353 |
|  | M+S+K+I | 0.0000 | 0.3333 | 0.4545 | 0.2286 |
|  | M+S+K+P | 0.1143 | 0.3030 | 0.3636 | 0.2571 |
|  | M+S+K+Y | 0.0571 | 0.2727 | 0.2727 | 0.1429 |
|  | M+S+P+I | 0.0000 | 0.3030 | 0.7576 | 0.2941 |
|  | M+S+Y+I | 0.0857 | 0.3333 | 0.4848 | 0.2000 |
|  | M+S+Y+P | 0.0857 | 0.3333 | 0.5152 | 0.2571 |
|  | M+K+P+I | 0.0857 | 0.2121 | 0.7273 | **0.2941** |
|  | M+K+Y+I | 0.0286 | 0.2121 | 0.4545 | 0.2000 |
|  | M+K+Y+P | 0.0857 | 0.3030 | 0.3939 | 0.2000 |
|  | M+Y+P+I | 0.0000 | 0.3333 | **0.7879** | 0.2353 |
|  | S+K+P+I | 0.0857 | 0.2121 | 0.7576 | **0.2941** |
|  | S+K+Y+I | 0.0857 | 0.2424 | 0.4545 | 0.2000 |
|  | S+K+Y+P | 0.0857 | 0.2727 | 0.5152 | 0.2571 |
|  | S+Y+P+I | 0.0286 | 0.2121 | **0.7879** | 0.2647 |
|  | K+Y+P+I | 0.0571 | 0.3030 | 0.7576 | 0.2647 |
| 5-barcodes | A+M+S+K+I | 0.0286 | **0.3939** | 0.5152 | 0.2286 |
|  | A+M+S+K+P | 0.0857 | 0.3030 | 0.3333 | 0.2059 |
|  | A+M+S+K+Y | 0.0286 | 0.3333 | 0.3030 | 0.1714 |
|  | A+M+S+P+I | 0.0857 | 0.3636 | 0.7879 | 0.2727 |
|  | A+M+S+Y+I | 0.1143 | 0.3636 | 0.4848 | 0.2571 |
|  | A+M+S+Y+P | **0.1429** | 0.3030 | 0.3939 | 0.2353 |
|  | A+M+K+P+I | 0.0571 | 0.3333 | 0.7273 | 0.2647 |
|  | A+M+K+Y+I | 0.1143 | 0.3030 | 0.4545 | 0.2000 |
|  | A+M+K+Y+P | 0.0857 | 0.3333 | 0.3939 | 0.2286 |
|  | A+M+Y+P+I | 0.0857 | 0.3030 | **0.8182** | 0.2647 |
|  | A+S+K+P+I | 0.0571 | 0.3636 | 0.6667 | 0.2727 |
|  | A+S+K+Y+I | 0.0286 | 0.3636 | 0.5758 | 0.1714 |
|  | A+S+K+Y+P | 0.0286 | 0.3333 | 0.4545 | 0.2353 |
|  | A+S+Y+P+I | 0.1143 | 0.3333 | 0.7879 | 0.2424 |
|  | A+K+Y+P+I | 0.0857 | 0.2727 | 0.7879 | 0.2353 |
|  | M+S+K+P+I | 0.0000 | 0.2424 | **0.8182** | **0.2941** |
|  | M+S+K+Y+I | 0.1143 | 0.3030 | 0.4545 | 0.2000 |
|  | M+S+K+Y+P | 0.0857 | 0.3333 | 0.4545 | 0.2571 |
|  | M+S+Y+P+I | 0.0571 | 0.3333 | 0.7879 | 0.2647 |
|  | M+K+Y+P+I | 0.1143 | 0.3030 | **0.8182** | 0.2353 |
|  | S+K+Y+P+I | 0.0857 | 0.3333 | 0.7879 | **0.2941** |
| 6-barcodes | A+M+S+K+P+I | **0.0857** | **0.3636** | 0.7576 | 0.2727 |
|  | A+M+S+K+Y+I | **0.0857** | **0.3636** | 0.5455 | 0.2000 |
|  | A+M+S+K+Y+P | **0.0857** | 0.3333 | 0.5758 | 0.2286 |
|  | A+M+S+Y+P+I | **0.0857** | **0.3636** | 0.7879 | **0.3030** |
|  | A+M+K+Y+P+I | 0.0000 | 0.3333 | **0.8485** | 0.2353 |
|  | A+S+K+Y+P+I | 0.0286 | 0.3333 | 0.8182 | 0.2424 |
|  | M+S+K+Y+P+I | 0.0000 | 0.3333 | 0.8182 | 0.2647 |
| 7-barcodes | A+M+S+K+Y+I+P | 0.0000 | 0.3636 | 0.8485 | 0.2424 |
| A: *psb*A-*trn*H; M: *mat*K-*trn*K; S: *ycf*3-*trn*S; K: *mat*K; Y: *ycf*1; I: ITS; P: SAP | | | |  |  |
| The highest species resolutions of each barcode and their possible combinations based on the four barcoding methods are shown in bold | | | | | |
