# Supplementary material for: Development of Chloroplast and Nuclear DNA Markers for Chinese Oaks (Quercus Subgenus Quercus) and Assessment of Their Utility as DNA Barcodes
Source: Front Plant Sci. 2017 May 19;8:816. doi: 10.3389/fpls.2017.00816 (PMC5437370; doi:10.3389/fpls.2017.00816)
Supplement: Note S1 — Detailed methods for data filtering of the ITS sequence. [file DataSheet1.DOCX]

**Note S1 Detailed methods for data filtering of the ITS sequence**

***Introduction***

The biparental inter transcribed spacer (ITS, including ITS1, 5.8S rDNA and ITS2) region is frequently used to estimate phylogenetic relationships at low taxonomic level (Harpke and Peterson, 2008). For many eukaryotes, the ITS region can be directly sequenced through traditional PCR approach, and generates single dominant sequences due homogenization process of this region, which may provide insights into species classification, hybridization, speciation, as well as intra- and interspecies diversity (Hribova *et al.*, 2011). However, for some closely related species that frequently hybridize, such as oaks of *Quercus*, concerted evolution of the ITS region may be impacted by past hybridization events and nucleolar dominance. The non-concerted evolution of ITS region may predate species divergence by introducing multiple copies of nucleolus organizer region (putative pseudogenes) and provide false phylogenetic signals among closely related species, although some of putative non-functional ITS copies were suggested to be useful for phylogenetic reconstructions as well as the functional ITS copies (Muir *et al.*, 2001; Zheng *et al.*, 2008).

In many species, concerted evolution will prevent sequence divergence among the rDNA families located at different chromosomal positions. While different types of paralogous and orthologous rDNA sequences may be present in the genome if the concerted evolution is incomplete, including the non-functional sequences referred as pseudogenes (Muir *et al.*, 2001). Thus, it is necessary to identify putative pseudogenous ITS sequences before species classification and phylogenetic reconstruction. Among the ITS region, the ITS2 spacer and 5.8S rDNA are much conserved in contrast to the ITS1 spacer within plants. For example, secondary structure of the ITS2 spacer includes four helices with typical pyrimidine-pyrimidine bulge in helix II and a conserved sequence region comprises TGGT in helix III (Hribova *et al.*, 2011). While the 5.8S rDNA contains three conserved motifs that are essential for identifying the functionality of ITS copies (Harpke and Peterson, 2008). The conserved secondary structures of ITS2 and 5.8S rDNA regions are recognized as useful method of detecting functional orthologues of ITS sequence.

Sequence data of the ITS region have been used to assess phylogenetic relationships of (some) oaks in *Quercus*. Most of these studies supported the traditional classification of several monophyletic groups in *Quercus* (i.e., Groups *Quercus*, *Cerris* and *Ilex*) (Manos *et al.*, 2001; Cavender-Bares *et al.*, 2004; Bellarosa *et al.*, 2005; Denk and Grimm, 2010; Simeone *et al.*, 2013), which in contrast to an early conflict (Samuel *et al.*, 1998), suggesting that the ITS pseudogenes could generate spurious results for phylogeny of oak species. For *Quercus*, clone is an ideal approach to obtain functional copies of ITS (Manos *et al.*, 1999; Denk and Grimm, 2010). Besides, with the improvement of high quality sequencing technology, functional orthologues of ITS can be amplified and sequenced directly through modified polymerase chain reaction (PCR) method (Zheng *et al.*, 2008). The latter, combined with subsequent filtration of potential pseudogene using the methods above, have been adopted in some phylogenetic studies on oak species, and showed comparable results to previous infrageneric relationships of *Quercus* (Manos *et al.*, 2001; Cavender-Bares *et al.*, 2004; Bellarosa *et al.*, 2005; Simeone *et al.*, 2013). Here, in this study, the ITS region of the Chinese oak species in subgenus *Quercus* were bidirectional sequenced using newly designed primers and modified PCR conditions. Potential representatives of pseudogenes were carefully examined and filtered with multiple methods for species identification work.

***Methods***

PCR approach of the functional ITS copies was modified (i.e., adding 4% DMSO to increase the specificity of functional copies during PCR) according to Zheng *et al.* (2008) using the newly designed primer pairs showed in the main text, which amplified partial ITS1 + 5.8S rDNA + partial ITS2 spacer. Clear single band of the PCR productions was visualized on 1% agarose gel, and was then purified and bidirectional sequenced by Sangon Biotech (Shanghai, China). Failures having overlap peaks in sequencing results were amplified at least three times to ensure multiple copies of ITS region amplified in the productions, and were removed from further analysis. Successful results of the ITS sequences were checked by eye using BioEdit 7.0.9.0 (Hall, 1999). Sequences having more than five continuous heterozygous sites were regarded as potential pseudogenes, while other heterozygous sites in functional ITS copies were coded using the IUPAC (International Union of Pure and Applied Chemistry) method.

The boundaries of the ITS region were determined and separated by comparison with some published ITS sequences obtained from Denk and Grimm (2010). Standard descriptive parameters of each sequence (i.e., sequence length, percentage of G + C content and pairwise sequence divergence within species) were estimated in BioEdit 7.0.9.0. Predicted secondary structures of the ITS2 spacer as well as the 5.8S rDNA region, and associated free energy values were evaluated using the minimum free energy algorithm and the latest free energy rules with the mfold v3.1 Web Server ([http://unafold.rna.albany.edu/?q=mfold](http://unafold.rna.albany.edu/?q=mfold/RNA-Folding-Form)). Folding temperature was fixed at 37°C with the maximum interior loop size of 30, while other parameters were kept as default. The minimum free energy (ΔG) of ITS2 and 5.8S rDNA for each sequence was estimated, and the four conserved helix structures as well as the pyrimidine-pyrimidine bulge in helix II (Hribova *et al.*, 2011) were tested for the ITS2 spacer. Additionally, the three conserved 5.8S motifs (CGATGAAGAACGTAGC, GAATTGCAGAATCC, and TTTGAAyGCA) in plants (Harpke and Peterson, 2008) were checked in the alignments of 5.8S rDNA sequences.

Finally, the retained sequences were filtered using a blast search method against the nucleotide database in the National Center for Biotechnology Information (NCBI), as well as a combined local database including functional ITS orthologues of some western Eurasian oak species through clone sequencing (Denk and Grimm, 2010) and a number of putative ITS pseudogenes of *Quercus* (Mayol and Rossello, 2001), to filter individuals that represent potential pseudogenous sequences.

***Results***

Using the modified PCR approach and newly designed primer pairs of ITS region, all individuals of the 35 Chinese oaks in subg. *Quercus* were successfully amplified and recovered by a clear single band of about 550 bp on the agarose gel. After sequencing of the PCR amplification, fourteen individuals (three of *Quercus spinosa*, one of *Q. pseudosemecarpifolia*, one of *Q. monimotricha*, two of *Q. senescens*, one of *Q. franchetii*, one of *Q. acrodonta*, one of *Q. baronii*, one of *Q. chenii*, one of *Q. alinea*, one of *Q. fabri*, and one of *Q. stewardii*) showed large overlaps in the sequencing results were removed due multiple copies of ITS region amplified. Additionally, two samples of *Q. spinosa* had indels (> 5 bp) in the conserved 5.8S rDNA region were also regarded as pseudogenes, and were deleted from the alignments (Table SS1). The aligned length of the ITS region (including indels) was 417 bp, which comprised partial ITS1 (81 to 83 bp), complete 5.8S rDNA (164 bp) and partial ITS2 spacer (171 to 178 bp). Note that the sizes of the 5.8S rDNA of the retained sequences were highly identical, which fit within the known ranges (163 to 164 bp) of 5.8S rDNA for the Angiosperm (Baldwin *et al.*, 1995), and are also consistent with the range size of clonal ITS of the western Eurasian oaks obtained from Denk and Grimm (2010). Total percentage of the GC content of the retained ITS sequences ranged from 58.53% to 62.17% among the 35 Chinese oaks. The percentage of the GC content of the (partial) ITS2 spacer extended from 63.07% to 70.62%, while the 5.8S rDNA region ranged from 51.83% to 53.66% among the 35 oak species (Table SS1). For the ITS alignment, the maximum sequence divergence of individual species ranged from 0 to 0.043 with an average value of 0.015. Two evergreen oaks, *Q. gilliana* (three samples) and *Q. phillyraeoides* (six individuals) showed no intraspecific variations based on the ITS region. While the deciduous oak species *Q. griffithii* had the highest sequence divergence (0.043) among its six individuals for the ITS. Range values of the maximum intraspecific divergence of the 35 Chinese oaks are similar to those detected in some Italian *Quercus* spp. (Bellarosa *et al.*, 2005), and are much lower than the divergence (> 0.1) of some oaks that were reported with putative pseudogenes of the ITS (Samuel *et al.*, 1998).

The secondary structure of the retained ITS sequences showed a high degree of stability among the 35 Chinese oak species that the mean minimum free energy (ΔG) ranged from -69.90 to -86.40 kcal/mol for ITS2, and from -47.50 to -50.70 kcal/mol for 5.8S rDNA at 37°C, respectively (Table SS1). Predicted structures of the (partial) ITS2 region revealed highly uniform foldings for all individuals of the 35 Chinese oaks, and we showed the putative foldings of five oak species (*Q. aliena*, *Q. acutissima*, *Q. spinosa*, *Q. engleriana* and *Q. baronii*) representing the five morphology-based sections (Fig. SS1). Within the secondary structures of the ITS2 region, the four conserved helix domains as well as the pyrimidine-pyrimidine bulge in Helix II were found in all individual sequences. The predicted structures of the conserved 5.8S rDNA for all retained sequences were also identical (Fig. SS1), and showed similar structure to a predicted folding for some sclerophyllous alpine oaks reported by Ma (2006), although the latter showed a larger size (173 bp) of the conserved 5.8S rDNA region than our results. In addition, the three conserved motifs in 5.8S rDNA of plants were located within the 5.8S rDNA region of all sequences of the 35 Chinese oak species.

The blast search of the retained ITS sequences in this study highly hit the homologous region of ITS in NCBI that were obtained through clone protocol and submitted by Manos *et al.* (1999) and Denk and Grimm (2010) with E-value < 0.0001. Additionally, when the ITS sequences of the 35 Chinese oaks were blasted against the local database comprising functional ITS copies and putative pseudogenous sequences, the results suggested more similarity of the retained ITS sequences to the functional ITS copies than to the putative pseudogenes.

Overall, the modified PCR approach with newly designed marker of the ITS region allows us to achieve a high rate (93%) of functional ITS copies for the Chinese oak species in subg. *Quercus*. These sequences were examined and filtered with multiple methods to ensure functional ITS copies. The estimated sequence parameters and secondary structures (i.e., GC content, intraspecific sequence divergence, length of the 5.8S rDNA and minimum free energy) of these ITS sequences (Table SS1) suggested conserved features within and among species/groups tested in this study. The prediction of secondary structures for ITS2 and 5.8S rDNA showed identical results among individuals of the 35 Chinese oaks (Fig. SS1), and were in agreement with previous predictions of the two conserved regions (ITS2 and 5.8S rDNA) from functional ITS copies (Bellarosa *et al.*, 2005). Moreover, a Blast work was performed against the web resource of ITS in NCBI and a local database including pervious published ITS sequences. All results highly supported that the retained 201 ITS sequences of the 35 Chinese oak species in this study were functional ITS orthologues, which can be used for species identification and phylogenetic analysis.

| **Table SS1** Estimated sequence parameters and secondary structures of ITS sequences obtained for 35 Chinese oak species | | | | | | |  |  |  | |  |  |  |
| --- | --- | --- | --- | --- | --- | --- | --- | --- | --- | --- | --- | --- | --- |
|  |  |  |  |  |  | ITS2 (partial) |  |  |  | | 5.8s |  |  |
| Group | Species | No. individuals | No. pseudogenes | Total G+C % | Maximum sequence divergence | G+C % | Four-helix model | Mean ΔG (kcal/mol) | | Length | | G+C% | Mean ΔG (kcal/mol) |
| *Ilex* | *Q. spinosa* | 15 | 5 | 61.37~62.09 | 0.015 | 69.32~70.45 | Yes | -79.90 |  | | 164 | 53.66 | -47.50 |
|  | *Q. aquifolioides* | 8 | 0 | 61.37~62.09 | 0.017 | 68.75~70.45 | Yes | -81.90 |  | | 164 | 53.66 | -47.50 |
|  | *Q. rehderiana* | 3 | 0 | 62.09~62.17 | 0.005 | 70.45~70.62 | Yes | -83.00 |  | | 164 | 53.66 | -47.50 |
|  | *Q. pseudosemecarpifolia* | 6 | 1 | 60.90~61.61 | 0.012 | 68.75~69.32 | Yes | -81.90 |  | | 164 | 53.66 | -47.50 |
|  | *Q. pannosa* | 4 | 0 | 61.37~61.85 | 0.008 | 68.75~69.32 | Yes | -81.90 |  | | 164 | 53.66 | -47.50 |
|  | *Q. longispica* | 6 | 0 | 61.61~61.85 | 0.010 | 68.75~69.32 | Yes | -81.90 |  | | 164 | 53.66 | -47.50 |
|  | *Q. monimotricha* | 4 | 1 | 61.85~62.09 | 0.005 | 69.32 | Yes | -81.90 |  | | 164 | 53.66 | -47.50 |
|  | *Q. senescens* | 10 | 2 | 61.61~62.09 | 0.008 | 69.32 | Yes | -81.90 |  | | 164 | 53.66 | -47.50 |
|  | *Q. guajavifolia* | 8 | 0 | 61.37~61.85 | 0.010 | 68.75~69.32 | Yes | -81.90 |  | | 164 | 53.66 | -47.50 |
|  | *Q. semecarpifolia* | 3 | 0 | 61.37 | 0.003 | 68.75 | Yes | -81.90 |  | | 164 | 53.66 | -47.50 |
|  | *Q. gilliana* | 3 | 0 | 61.61 | 0.000 | 68.75 | Yes | -83.30 |  | | 164 | 53.66 | -47.50 |
|  | *Q. engleriana* | 3 | 0 | 60.81~61.14 | 0.010 | 66.86~68.18 | Yes | -81.70 |  | | 164 | 53.66 | -47.50 |
|  | *Q. cocciferoides* | 3 | 0 | 60.81~61.05 | 0.008 | 66.86~67.43 | Yes | -74.70 |  | | 164 | 53.66 | -47.50 |
|  | *Q. phillyraeoides* | 6 | 0 | 61.05 | 0.000 | 68.00 | Yes | -82.40 |  | | 164 | 53.66 | -47.00 |
|  | *Q. franchetii* | 3 | 1 | 60.33~61.85 | 0.017 | 66.29~68.75 | Yes | -86.40 |  | | 164 | 54.27~53.66 | -47.50 |
|  | *Q. acrodonta* | 6 | 1 | 60.57~61.28 | 0.008 | 66.86~68.57 | Yes | -81.30 |  | | 164 | 53.66 | -47.00 |
|  | *Q. lanata* | 3 | 0 | 61.14~61.70 | 0.015 | 68.18~68.93 | Yes | -82.30 |  | | 164 | 53.66 | -47.50 |
|  | *Q. tarokoensis* | 4 | 0 | 61.61~62.03 | 0.008 | 68.75~69.66 | Yes | -72.30 |  | | 164 | 53.66 | -47.50 |
|  | *Q. dolicholepis* | 6 | 0 | 60.33~61.05 | 0.010 | 66.29~68.00 | Yes | -82.40 |  | | 164 | 53.66 | -47.00 |
|  | *Q. oxyphylla* | 6 | 0 | 60.19~61.05 | 0.017 | 65.91~68.00 | Yes | -82.40 |  | | 164 | 53.05~53.66 | -47.50 |
|  | *Q. baronii* | 9 | 1 | 59.95~60.28 | 0.008 | 65.91~66.10 | Yes | -84.20 |  | | 164 | 53.05~53.66 | -47.50 |
| *Cerris* | *Q. acutissima* | 9 | 0 | 60.19~61.39 | 0.012 | 66.08~69.01 | Yes | -85.10 |  | | 164 | 53.66 | -47.50 |
|  | *Q. variabilis* | 6 | 0 | 60.67~61.15 | 0.010 | 67.25~68.42 | Yes | -85.40 |  | | 164 | 53.66 | -47.50 |
|  | *Q. chenii* | 3 | 1 | 60.67~60.91 | 0.003 | 66.67~67.25 | Yes | -78.80 |  | | 164 | 53.66 | -47.50 |
| *Quercus* | *Q. aliena* | 9 | 1 | 58.77~60.57 | 0.036 | 63.64~66.86 | Yes | -79.80 |  | | 164 | 51.83~53.66 | -47.50 |
|  | *Q. aliena* var. *acuteserrata* | 9 | 0 | 59.48~60.66 | 0.029 | 64.77~67.05 | Yes | -78.90 |  | | 164 | 53.05~53.66 | -47.50 |
|  | *Q. dentata* | 9 | 0 | 59.86~60.57 | 0.024 | 65.14~66.86 | Yes | -84.80 |  | | 164 | 53.05~53.66 | -50.70 |
|  | *Q. fabri* | 9 | 1 | 58.53~60.57 | 0.034 | 63.07~66.86 | Yes | -79.40 |  | | 164 | 51.83~53.66 | -47.50 |
|  | *Q. serrata* | 8 | 0 | 59.86~60.43 | 0.019 | 65.14~66.86 | Yes | -74.40 |  | | 164 | 53.66 | -47.50 |
|  | *Q. serrata* var. *brevipetiolata* | 6 | 0 | 59.86~60.57 | 0.024 | 65.71~66.86 | Yes | -81.30 |  | | 164 | 53.05~53.66 | -50.70 |
|  | *Q. liaotungensis* | 9 | 0 | 59.48~60.33 | 0.029 | 64.20~66.29 | Yes | -79.70 |  | | 164 | 53.05~53.66 | -47.50 |
|  | *Q. mongolica* | 9 | 0 | 59.24~60.43 | 0.029 | 63.64~66.29 | Yes | -74.80 |  | | 164 | 53.05~53.66 | -50.70 |
|  | *Q. griffithii* | 6 | 0 | 58.53~60.19 | 0.043 | 63.07~66.48 | Yes | -69.90 |  | | 164 | 51.83~53.66 | -47.50 |
|  | *Q. yunnanensis* | 3 | 0 | 59.48~60.57 | 0.027 | 64.77~66.86 | Yes | -76.50 |  | | 164 | 53.05~53.66 | -47.50 |
|  | *Q. stewardii* | 3 | 1 | 59.24 | 0.029 | 63.07~64.77 | Yes | -82.10 |  | | 164 | 53.05~53.66 | -47.50 |


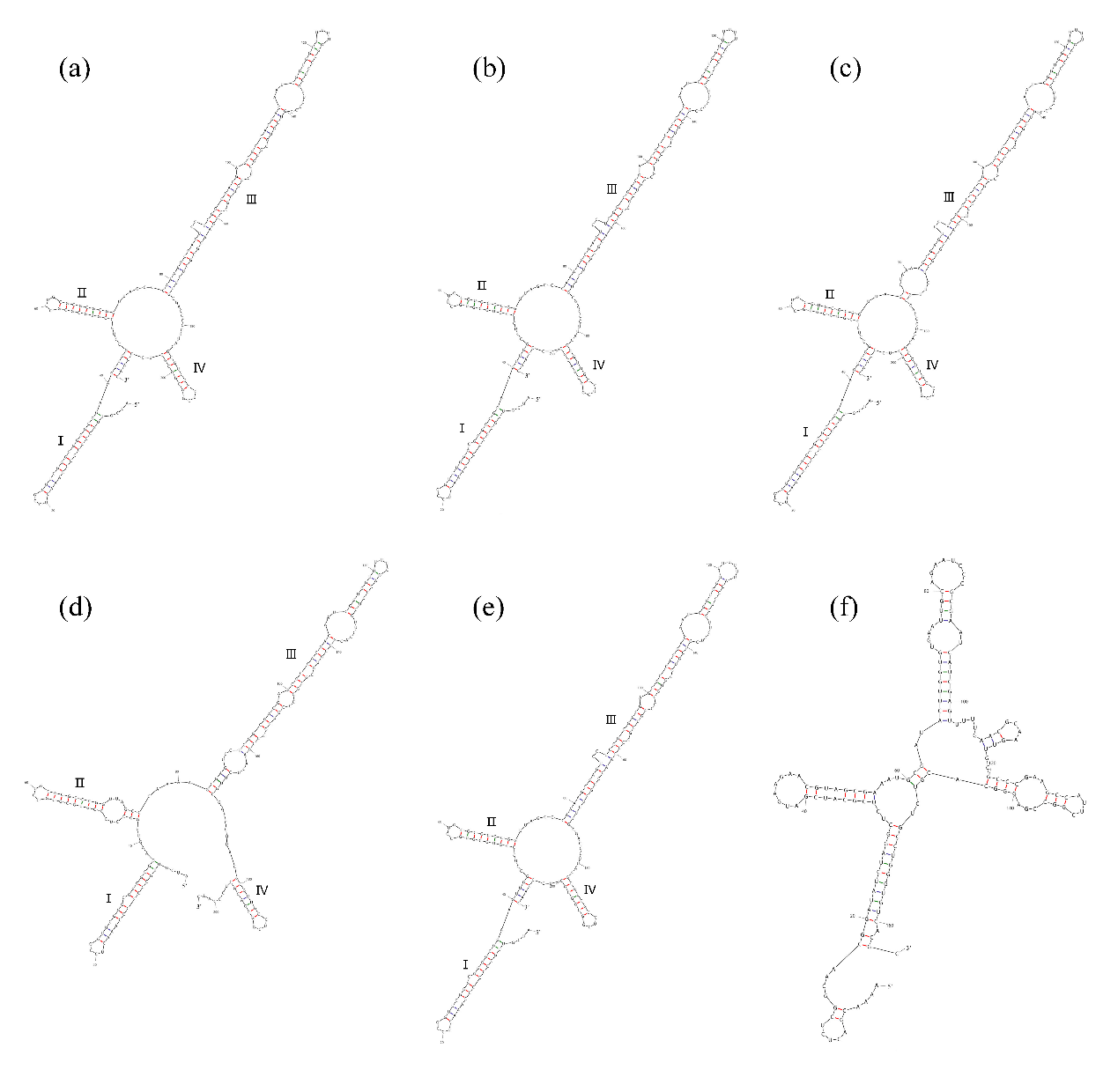


**Figure SS1** Putative secondary structures of the ITS2 region for (a) *Q. spinosa*, (b) *Q. engleriana,* (c) *Q. baronii*, (d) *Q. acutissima*, and (e) *Q. aliena* representing the five morphology-based sections of the Chinese oak species in subgenus *Quercus*; (f) shows the predicted secondary structure of the 5.8S rDNA region for *Q. dentata*.

***References***

Baldwin, B.G., Sanderson, M.J., Porter, J.M., Wojciechowski, M.F., Campbell, C.S., Donoghue, M.J. (1995). The ITS region of nuclear ribosomal DNA: a valuable source of evidence on Angiosperm phylogeny. *Ann. Mo. Bot. Gard.* 82 (2): 247-277

Bellarosa, R., Simeone, M.C., Papini, A., Schirone, B. (2005). Utility of ITS sequence data for phylogenetic reconstruction of Italian *Quercus* spp. *Mol. Phylogenet. Evol.* 34: 355-370

Cavender-Bares, J., Ackerly, D.D., Baum, D.A., Bazzaz, F.A. (2004). Phylogenetic Overdispersion in Floridian Oak Communities. *Am. Nat.* 163 (6): 823-842

Denk, T., and Grimm, G.W. (2010). The oaks of western Eurasia: Traditional classifications and evidences from two nuclear markers. *TAXON* 59 (2): 351-366

Hall, T.A. (1999). BioEdit: a user-friendly biological sequence alignment editor and analysis program for Windows 95/98/NT. *Nucleic Acids Symp. Ser.* 41: 95-98

Harpke, D., and Peterson, A. (2008). 5.8S motifs for the identification of pseudogenic ITS regions. *Botany* 86: 300-305

Hribova, E., Cizkova, J., Christelova, P., Taudien, S., de Langhe, E., Dolezel, J. (2011). The ITS1-5.8S-ITS2 Sequence Region in the Musaceae: Structure, Diversity and Use in Molecular Phylogeny. *PLoS One* 6 (3): e17863

Ma, C.L. (2006). Phylogeny and Biogeography of *Quercus* Sect. *Heterobalanus*. [Dissertation]. [Kunming (China)] Institute of Botany, The Chinese Academy of Sciences.

Manos, P.S., Doyle, J.J., and Nixon, K.C. (1999). Phylogeny, Biogeography, and Processes of Molecular Differentiation in *Quercus* Subgenus *Quercus* (Fagaceae). *Mol. Phylogenet. Evol.* 12 (3): 333-349

Manos, P.S., Zhou, Z.Z., and Cannon, C.H. (2001). Systematics of Fagaceae: Phylogenetic tests of reproductive trait evolution. *Int. J. Plant Sci.* 162 (6): 1361-1379

Mayol, M., and Rossello, J.A. (2001).Why Nuclear Ribosomal DNA Spacers (ITS) Tell Different Stories in *Quercus*. *Mol. Phylogenet. Evol.* 19 (2): 167-176

Muir, G., Fleming, C.C., and Schlotterer, C. (2001). Three Divergent rDNA Clusters Predate the Species Divergence in *Quercus petraea* (Matt.) Liebl. and *Quercus robur* L. *Mol. Biol. Evol*. 18 (2): 112-119

Samuel, R., Bachmair, A., Jobst, J., Ehrendorfer, F. (1998). ITS sequences from nuclear rDNA suggest unexpected phylogenetic relationships between Euro-Mediterranean, East Asiatic and North American taxa of *Quercus* (Fagaceae). *Plant Syst. Evol.* 211: 129-139

Simeone, M.C., Piredda, R., Papini, A., Vessella, F., Schirone, B. (2013). Application of plastid and nuclear markers to DNA barcoding of Euro-Mediterranean oaks (*Quercus*, Fagaceae): problems, prospects and phylogenetic implications. *Bot. J. Linn. Soc.* 172: 478-499

Zheng, X.Y., Cai, D.Y., Yao, L.H., Teng, Y.W. (2008). Non-concerted ITS evolution, early origin and phylogenetic utility of ITS pseudogenes in *Pyrus*. *Mol. Phylogenet. Evol.* 48: 892-903
