# Supplementary material for: Development of Chloroplast and Nuclear DNA Markers for Chinese Oaks (Quercus Subgenus Quercus) and Assessment of Their Utility as DNA Barcodes
Source: Front Plant Sci. 2017 May 19;8:816. doi: 10.3389/fpls.2017.00816 (PMC5437370; doi:10.3389/fpls.2017.00816)
Supplement: Figure S10 — NJ tree of the single-copy nuclear gene (SAP) for the Chinese oak species in Quercus subgenus Quercus highlights the species discrimination of Quercus acutissima from Quercus variabilis + Quercus chenii in Group Cerris. [file Image10.PDF]

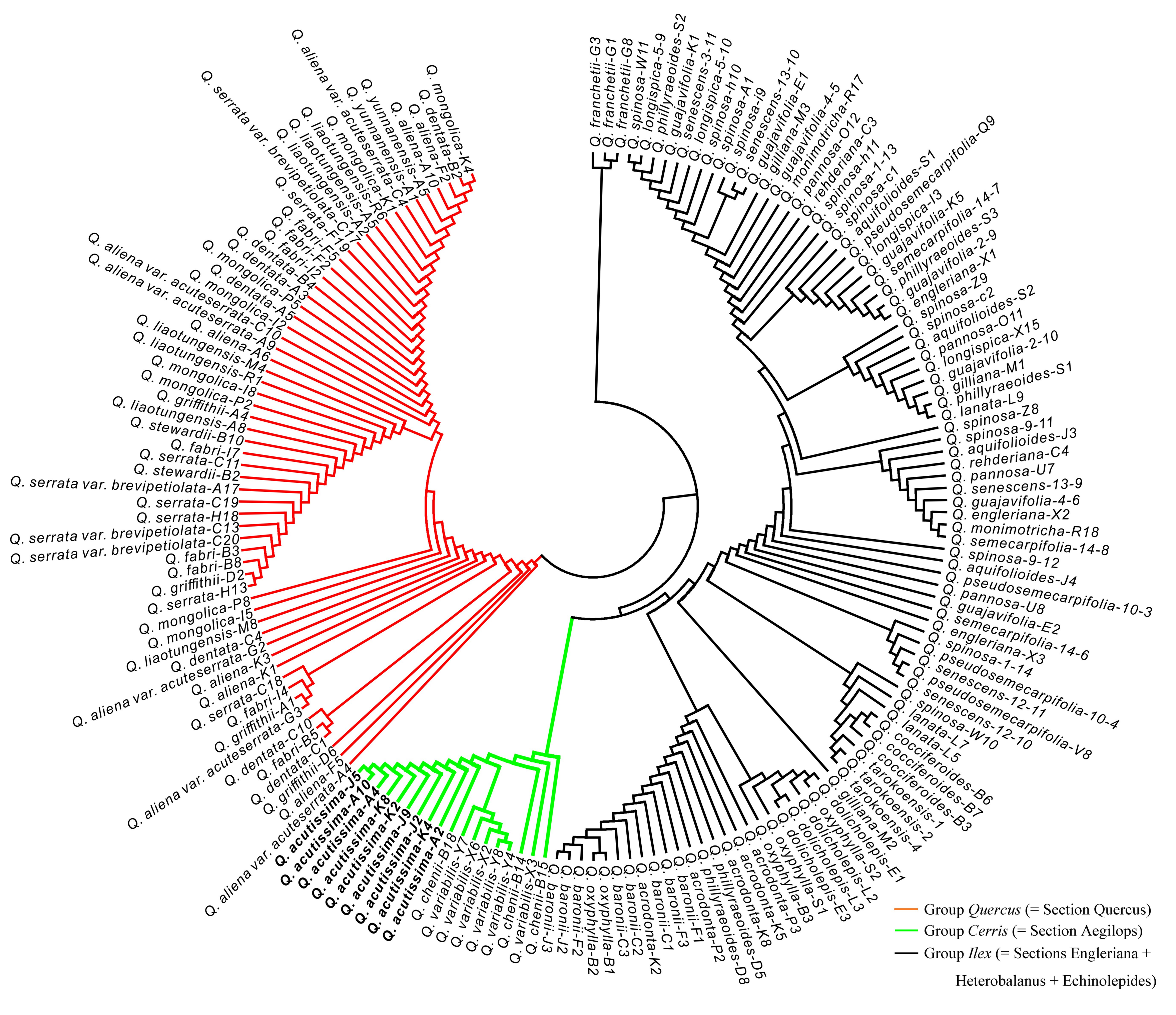

Q. franchetii-G3
Q. franchetii-G1
Q. franchetii-G8
Q. spinosa-W11
Q. longispica-5-9
Q. phillyraeoides-S2
Q. guajavifolia-K1
Q. senescens-3-11
Q. longispica-5-10
Q. spinosa-h10
Q. spinosa-A1
Q. spinosa-i9
Q. senescens-13-10
Q. guajavifolia-E1
Q. gilliana-M3
Q. guajavifolia-4-5
Q. monimotricha-R17
Q. pannosa-O12
Q. rehderiana-C3
Q. spinosa-h11
Q. spinosa-1-13
Q. spinosa-c1
Q. aquifolioides-S1
Q. pseudosemecarpifolia-Q9
Q. longispica-I3
Q. guajavifolia-K5
Q. semecarpifolia-14-7
Q. phillyraeoides-S3
Q. guajavifolia-2-9
Q. engleriana-X1
Q. spinosa-Z9
Q. spinosa-c2
Q. aquifolioides-S2
Q. pannosa-O11
Q. longispica-X15
Q. guajavifolia-2-10
Q. gilliana-M1
Q. phillyraeoides-S1
Q. lanata-L9
Q. spinosa-Z8
Q. spinosa-9-11
Q. aquifolioides-J3
Q. rehderiana-C4
Q. pannosa-U7
Q. senescens-13-9
Q. guajavifolia-4-6
Q. engleriana-X2
Q. monimotricha-R18
Q. semecarpifolia-14-8
Q. spinosa-9-12
Q. aquifolioides-J4
Q. pseudosemecarpifolia-10-3
Q. pannosa-U8
Q. guajavifolia-E2
Q. semecarpifolia-14-6
Q. engleriana-X3
Q. spinosa-1-14
Q. pseudosemecarpifolia-10-4
Q. senescens-12-11
Q. pseudosemecarpifolia-V8
Q. senescens-12-10
Q. spinosa-W10
Q. lanata-L7
Q. lanata-L5
Q. cocciferoides-B6
Q. cocciferoides-B7
Q. cocciferoides-B3
Q. tarokoensis-1
Q. tarokoensis-2
Q. tarokoensis-4
Q. gilliana-M2
Q. dolicholepis-E1
Q. oxyphylla-L2
Q. dolicholepis-S2
Q. dolicholepis-L3
Q. dolicholepis-E3
Q. oxyphylla-S1
Q. oxyphylla-B3
Q. acrodonta-P3
Q. acrodonta-K5
Q. acrodonta-K8
Q. phillyraeoides-D5
Q. phillyraeoides-D8
Q. acrodonta-P2
Q. baronii-F1
Q. baronii-F3
Q. baronii-C1
Q. acrodonta-K2
Q. baronii-C2
Q. baronii-C3
Q. oxyphylla-B1
Q. oxyphylla-B2
Q. baronii-F2
Q. baronii-J2
Q. baronii-J3
Q. chenii-B15
Q. variabilis-X3
Q. chenii-B14
Q. variabilis-Y4
Q. variabilis-Y8
Q. variabilis-X2
Q. variabilis-X6
Q. variabilis-Y7
Q. chenii-B18
Q. acutissima-A2
Q. acutissima-K4
Q. acutissima-J2
Q. acutissima-J9
Q. acutissima-K2
Q. acutissima-K8
Q. acutissima-A4
Q. acutissima-A10
Q. acutissima-J5
Q. aliena var. acuteserrata-A4
Q. aliena-F5
Q. griffithii-D6
Q. dentata-C1
Q. fabri-B5
Q. dentata-C10
Q. aliena var. acuteserrata-G3
Q. griffithii-A1
Q. fabri-I4
Q. serrata-C18
Q. aliena-K1
Q. aliena-K3
Q. aliena var. acuteserrata-G2
Q. dentata-C4
Q. liaotungensis-M8
Q. mongolica-I5
Q. mongolica-P8
Q. serrata-H13
Q. griffithii-D2
Q. fabri-B8
Q. fabri-B3
Q. serrata var. brevipetiolata-C20
Q. serrata var. brevipetiolata-C13
Q. serrata-H18
Q. serrata-C19
Q. serrata var. brevipetiolata-A17
Q. stewardii-B2
Q. serrata-C11
Q. fabri-I7
Q. stewardii-B10
Q. liaotungensis-A8
Q. griffithii-A4
Q. mongolica-P2
Q. mongolica-I8
Q. liaotungensis-R1
Q. liaotungensis-M4
Q. aliena-A6
Q. aliena var. acuteserrata-A9
Q. aliena var. acuteserrata-C10
Q. mongolica-I2
Q. dentata-A5
Q. mongolica-P5
Q. dentata-A3
Q. dentata-B4
Q. fabri-I2
Q. fabri-F2
Q. fabri-F5
Q. serrata-F19
Q. serrata var. brevipetiolata-C17
Q. liaotungensis-A2
Q. liaotungensis-A5
Q. liaotungensis-R6
Q. mongolica-K1
Q. aliena var. acuteserrata-C4
Q. yunnanensis-A1
Q. yunnanensis-A5
Q. aliena-A10
Q. aliena-F2
Q. dentata-B2
Q. mongolica-K4
Group *Quercus* (= Section Quercus)
Group *Cerris* (= Section Aegilops)
Group *Ilex* (= Sections Engleriana + Heterobalanus + Echinolepides)
